# Regulation of the Muscarinic M_3_ Receptor by Myocardin-Related Transcription Factors

**DOI:** 10.3389/fphys.2021.710968

**Published:** 2021-09-03

**Authors:** Li Liu, Catarina Rippe, Ola Hansson, Dmytro Kryvokhyzha, Steven Fisher, Mari Ekman, Karl Swärd

**Affiliations:** ^1^Department of Experimental Medical Science, Lund, Sweden; ^2^Department of Urology, Qingyuan People's Hospital, The Sixth Affiliated Hospital of Guangzhou Medical University, Qingyuan, China; ^3^Department of Clinical Sciences, Lund University Diabetes Centre, Malmö, Sweden; ^4^Institute for Molecular Medicine Finland (FIMM), Helsinki University, Helsinki, Finland; ^5^Department of Medicine (Cardiology) and Physiology and Biophysics, University of Maryland-Baltimore, Baltimore, MD, United States

**Keywords:** cholinergic neurotransmission, pharmacology, acetylcholine, signaling, vasodilatation

## Abstract

Myocardin-related transcription factors (MRTFs: myocardin/*MYOCD*, MRTF-A/*MRTFA*, and MRTF-B/*MRTFB*) are co-factors of serum response factor (SRF) that activate the smooth muscle cell (SMC) gene program and that play roles in cardiovascular development and mechanobiology. Gain and loss of function experiments have defined the SMC gene program under control of MRTFs, yet full understanding of their impact is lacking. In the present study, we tested the hypothesis that the muscarinic M_3_ receptor (*CHRM3*) is regulated by MRTFs together with SRF. Forced expression of MYOCD (8d) in human coronary artery (SMC) followed by RNA-sequencing showed increased levels of M_2_, M_3_, and M_5_ receptors (*CHRM2*: 2-fold, *CHRM3*: 16-fold, and *CHRM5*: 2-fold). The effect of MYOCD on M_3_ was confirmed by RT-qPCR using both coronary artery and urinary bladder SMCs, and correlation analyses using human transcriptomic datasets suggested that M_3_ may also be regulated by MRTF-B. Head-to-head comparisons of MYOCD, MRTF-A and MRTF-B, argued that while all MRTFs are effective, MRTF-B is the most powerful transactivator of *CHRM3*, causing a 600-fold increase at 120h. Accordingly, MRTF-B conferred responsiveness to the muscarinic agonist carbachol in Ca^2+^ imaging experiments. M_3_ was suppressed on treatment with the MRTF-SRF inhibitor CCG-1423 using SMCs transduced with either MRTF-A or MRTF-B and using intact mouse esophagus in culture (by 92±2%). Moreover, silencing of SRF with a short hairpin reduced *CHRM3* (by >60%) in parallel with α-actin (*ACTA2*). Tamoxifen inducible knockout of Srf in smooth muscle reduced *Srf* (by 54±4%) and *Chrm3* (by 41±6%) in the urinary bladder at 10days, but *Srf* was much less reduced or unchanged in aorta, ileum, colon, trachea, and esophagus. Longer induction (21d) further accentuated the reduction of *Chrm3* in the bladder and ileum, but no change was seen in the aorta. Single cell RNA-sequencing revealed that *Mrtfb* dominates in ECs, while *Myocd* dominates in SMCs, raising the possibility that *Chrm3* may be driven by Mrtfb-Srf in the endothelium and by Myocd-Srf in SMCs. These findings define a novel transcriptional control mechanism for muscarinic M_3_ receptors in human cells, and in mice, that could be targeted for therapy.

## Introduction

G protein-coupled receptors (GPCRs) represent the largest group of proteins targeted by clinical drugs, with well over 100 being affected by FDA-approved substances (Sriram and Insel, 2018). Among these are the muscarinic M_2_ (*CHRM2*) and M_3_ (*CHRM3*) receptors (Wess et al., 2007). Muscarinic receptors are expressed in the brain, on target organs of the parasympathetic nervous system, including pacemaking cells in the heart, smooth muscle cells (SMCs), glandular cells, and on endothelial cells (Caulfield, 1993; Wess et al., 2007). Agonists and antagonists of M_2_ and M_3_ are used in clinical conditions ranging from airway obstruction (chronic obstructive pulmonary disease) to bladder overactivity and glaucoma (Ritter et al., 2020). Anticholinesterases, which indirectly affect muscarinic receptors by inhibiting breakdown of acetylcholine, may additionally be of some utility in Alzheimer’s disease (Ritter et al., 2020).

M_2_ and M_3_ receptors are often co-expressed, and their physiological functions have been defined in mice using knockout strategies (Wess et al., 2007). M_3_ deficient mice, for example, have reduced body weights and impaired salivation, dilated pupils under bright light, and urinary retention caused by reduction of muscarinic contractility in the bladder (Matsui et al., 2000; Yamada et al., 2001). Moreover, both cholinergic vasodilatation (Gericke et al., 2011) and vasoconstriction (Gericke et al., 2014) are largely abolished. Dual knockout of M_2_ and M_3_ causes further impairment of gastrointestinal (Matsui et al., 2002) and airway (Struckmann et al., 2003) contractility compared to the isolated knockout of M_3_, but viability remains unaffected. In addition, knockout studies have defined roles of muscarinic receptors in neuronal activity and plasticity in the brain (Thomsen et al., 2018). In contrast to the wealth of knowledge generated on physiological functions of muscarinic receptor subtypes, there is a paucity of information regarding the transcriptional control of these receptors. This is a void of knowledge that needs to be filled because transcriptional control mechanisms could be suited for therapy.

Myocardin-related transcription factors (MRTFs: myocardin/*MYOCD*, MRTF-A/*MRTFA*, and MRTF-B/*MRTFB*) act together with the serum response factor (SRF) to drive a broad mesodermal gene program (Miano, 2003, 2015; Olson and Nordheim, 2010; Sward et al., 2016). MRTFs play important roles beyond development in tissues, such as the heart (Parlakian et al., 2005; Mokalled et al., 2015), smooth muscle (Huang et al., 2015), and endothelial cells (Weinl et al., 2013, 2015). A defining property of MRTFs is that they respond to mechanical signals, such as biomechanical force (Chan et al., 2010), stretch (Zhao et al., 2007; Cui et al., 2015), and matrix stiffness (Jain et al., 2013; Foster et al., 2017; Hadden et al., 2017), and the underlying mechanism likely involves actin dynamics (Zhao et al., 2007; Finch-Edmondson and Sudol, 2016). Numerous recent studies have cataloged genes that are activated by overexpression of wild type and constitutively active MRTFs using RNA-sequencing (Zhao et al., 2016; Kim et al., 2017; Hu et al., 2019), but GPCRs and ion channels are often underrepresented in such dataset (Miano et al., 2007), and many of these datasets have limited sample sizes. Polymerase chain reaction (PCR)-based studies with larger sample sizes have demonstrated that MRTFs may play a role for GPCR expression (Krawczyk et al., 2018).

**Figure 1:**
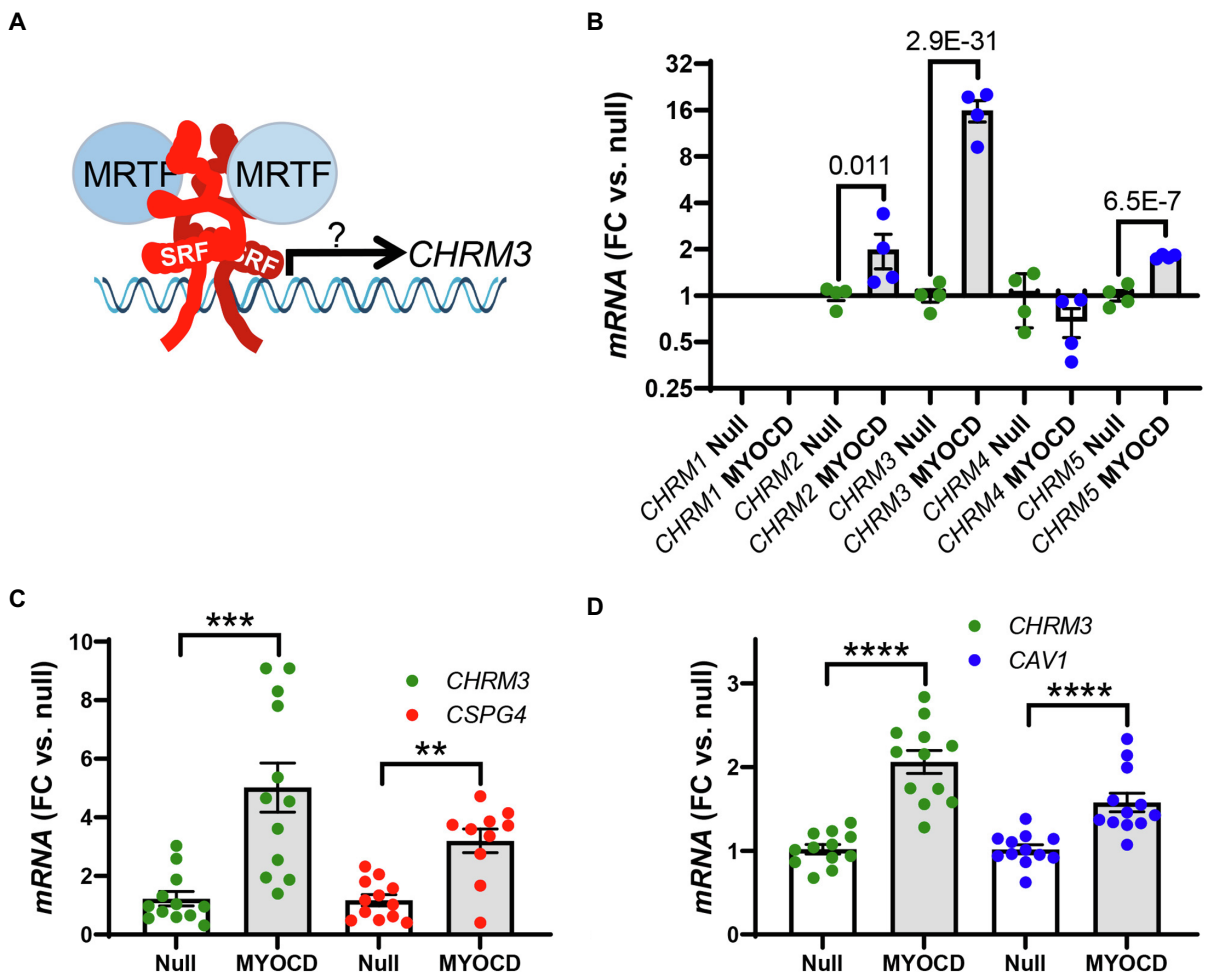
The transcriptional coactivator myocardin (MYOCD) regulates expression of the M_3_ muscarinic receptor (*CHRM3*). Panel **A** shows a schematic representation of the hypothesis that we set out to test in the present work, namely, that myocardin-related transcription factors (MRTFs) control expression of the M_3_ muscarinic receptor for acetylcholine (*CHRM3*). Panel **B** shows data from an RNA-sequencing experiment were MYOCD was overexpressed in cultured human coronary artery smooth muscle cells (hCASMCs) for 8days (n=4 null and 4 MYOCD). Ad-CMV-MYOCD was used for overexpression, and Ad-CMV-null virus at the same multiplicity of infection was used as control. The full dataset of differentially expressed genes is given in the Supplementary Material. Brackets in panel **B** show adjusted P-values for the indicated comparisons of M_2_ (*CHRM2*), M_3_ (*CHRM3*), and M_5_ (*CHRM5*) receptor transcripts between conditions. Panels **C** and **D** show confirmation using RT-qPCR (*n=*12 throughout) that overexpression of myocardin (96h) upregulates the M_3_ receptor transcript. Experiments were run using human coronary artery SMCs in **C** and using human bladder SMCs in **D**. *CSPG4* and *CAV1* were used as positive control targets in **C**, **D**. Bar graphs in this and the following figures show means±SEM, but individual data points are also given. ****p<0.0001, ***p<0.001 and **p<0.01.

In recent work, we characterized conditional and smooth muscle-specific knockouts of YAP and TAZ, which are coactivators of TEA domain transcription factors (TEADs), and uncovered a lethal colonic phenotype (Daoud et al., 2020). Like MRTFs, YAP and TAZ are mechano-activated (Hadden et al., 2017), and highly expressed in smooth muscle. YAP may act together with MRTFs, especially MRTF-B, and this synergy is governed by a direct physical interaction (Kim et al., 2017). Among transcripts that were reduced in the colon and urinary bladder of YAP/TAZ knockout mice were *Chrm2* and *Chrm3*. Other transcripts that were concordantly reduced were established target genes of MRTF-SRF signaling. Indeed, we found that knockout of YAP and TAZ also caused a parallel reduction of *Srf*. Together, these findings raise the possibility that *Chrm2* and *Chrm3* (M_2_ and M_3_) may be regulated by MRTF-SRF. The current study was initiated to address this hypothesis (depicted graphically in Figure 1A) and to fill the current gap of knowledge regarding transcriptional regulation of clinically relevant GPCRs.

## Materials and Methods

### Bulk RNA-Sequencing

RNA was prepared (miRNeasy, Qiagen) from human coronary artery SMCs transduced with either Ad-CMV-null or Ad-CMV-MYOCD virus (200 MOI, see below). RNA integrity was assessed using TapeStation (Agilent). Next, libraries were prepared using the TruSeq^®^ Stranded Total RNA Library Prep. For demultiplexing index, adapters were added (TruSeq RNA Single Indexes Set A,12 Indexes). Sequencing was performed using NextSeq 500/550 High Output Kit v2.5 on an Illumina NextSeq 500 instrument (75bp, paired end). Reads were mapped with STAR (Dobin et al., 2013) in 2-pass mode and counted with featureCounts (Liao et al., 2014). Normalization and differential gene expression analysis were performed using DESeq2 (Love et al., 2014). Qualities of sequences, alignments, and read counting were assessed with fastQC (De Sena Brandine and Smith, 2019), qualimap (Okonechnikov et al., 2016), and multiQC (Ewels et al., 2016). All the code, including a conda environment, Snakemake file, and R markdown notebooks, are available at https://github.com/LUDC-bioinformatics/SMC_MYOCD

### Cell Culture and Adenoviral Overexpression and Silencing

Human coronary artery SMCs were from Thermo Scientific/Gibco (C0175C) and cultured in medium 231 (M231500) with growth supplement (SMGS: S00725) and 50U/50μg/ml PEST (Biochrom, A2212). Human bladder smooth muscle cells (HBSMCs) were isolated from detrusor strips as described (Zhu et al., 2017). HBSMCs were cultured in DMEM/Ham’s F-12 medium with glutamine (Biochrom; FG4815), 10% fetal bovine serum (FBS; Biochrom; S0115), and 50U/50μg/ml PEST (Biochrom; A2212). Human coronary artery endothelial cells were obtained from Lonza (CC-2585) and cultured in EGM-2 MV Microvascular Endothelial Cell Growth Medium-2 BulletKit (CC-3202), which contains EBM-2 Basal Medium (CC-3156) and EGM-2 MV Microvascular Endothelial Cell Growth Medium SingleQuotsTM supplements (CC-4147). All primary cells were used in passages 3–8 and they were maintained in a standard cell culture incubator (37°C, 95% air, and 5% CO2).

**Figure 2:**
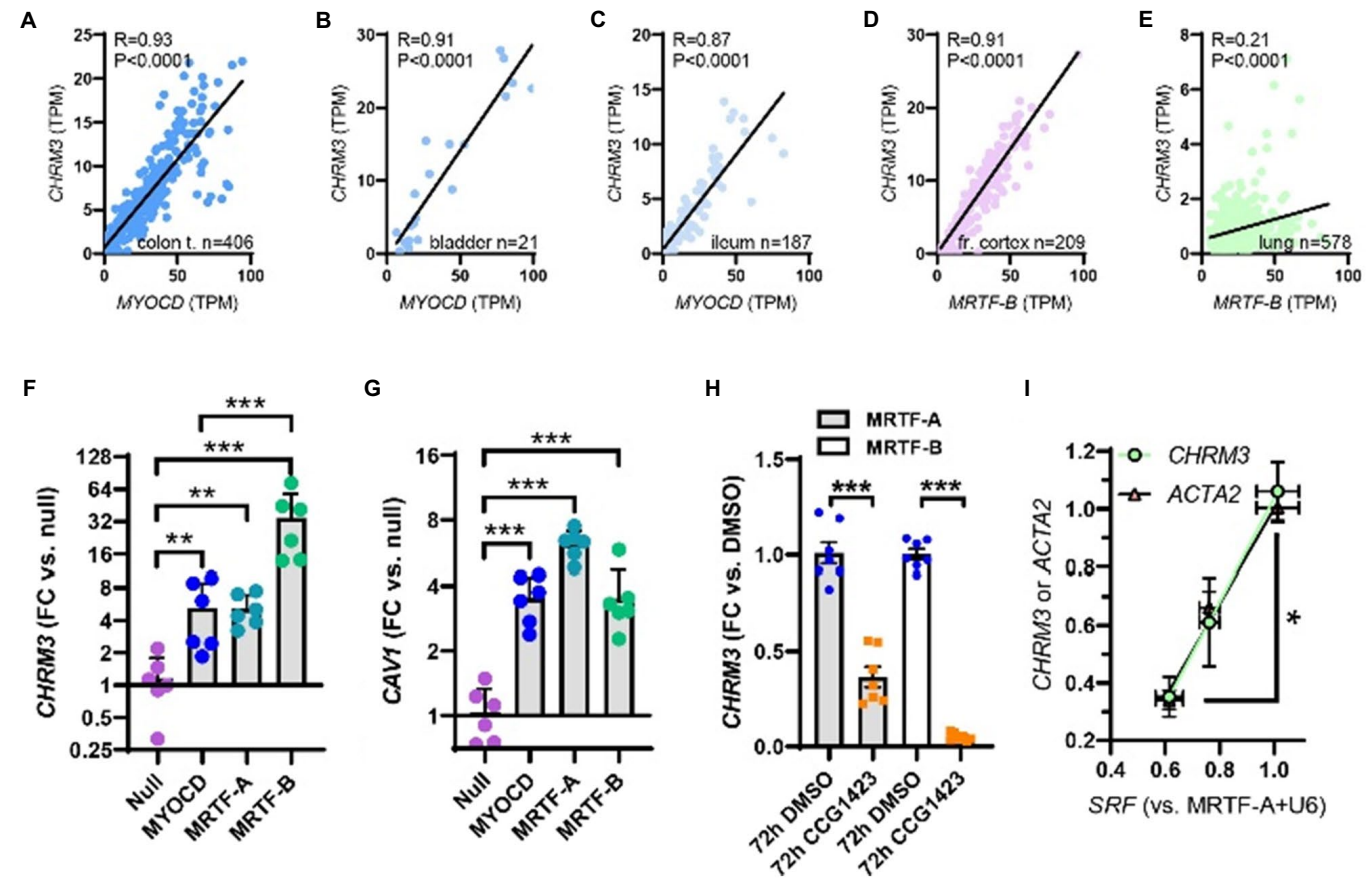
Myocardin (MYOCD) correlates with the M_3_ muscarinic receptor (*CHRM3*) across human tissues and SRF is critical for *CHRM3* regulation by MRTFs. **(A)** through **(C)** show correlations at the mRNA level of MYOCD vs. M_3_ (*CHRM3*) in the human gastrointestinal tract and urinary bladder. In brain **(D)** and lung **(E)**, *MRTFB*, rather than *MYOCD*, correlated with M_3_. This prompted us to examine if all MRTFs (MYOCD, MRTF-A, and MRTF-B) regulate M_3_ at the mRNA level. Viral overexpression in human coronary artery SMCs showed that MRTF-B was a more effective transactivator of *CHRM3* than MYOCD (**F**, *n=*6), despite having the same effect as MYOCD on another target (*CAV1*, **G**). **(H)** Shows reduction of *CHRM3* after treatment for 72h with the MRTF-SRF inhibitor CCG-1423 (10μM, *n=*6). Cells were transduced with either MRTF-A (gray bars) or MRTF-B (white bars). **(I)** Shows that knockdown of serum response factor (SRF, 0, 30, and 100 MOI of Ad-shSRF) reduces *CHRM3* (green/black circles) in parallel with *ACTA2* (pink/black triangles, *n=*4, per condition). MRTF-A was overexpressed throughout in **(I)**. ***p<0.001, **p<0.01, and *p<0.05.

Adenoviral vectors for overexpression and silencing were obtained from Vector Biolabs (Ad-h-MYOCD, ADV-216227; Ad-h-MKL1/eGFP, ADV-215499; Ad-h-MKL2, ADV-215500; Ad-CMV-Null, #1300; AD-h-YAP1, ADV-227945; Ad-h-shSRF, shADV-224,323; and Ad-GFP-U6-shRNA, #1122) and used at the indicated titers (multiplicities of infection, MOI). Here, Ad-CMV-Null, #1300 and Ad-GFP-U6-shRNA, #1122 were used as negative controls. Most transduced cells were harvested at 96h unless specified. For instance, in Figure 1B, the cells were collected on the eigth day after transduction, and in Figure 2, the transduction time was 120h.

### Cell Treatment

In Figure 2H, human coronary artery SMCs were transduced with MRTF-A virus or MRTF-B virus (200 MOI) in 1% Smooth Muscle Differentiation Supplement (SMDS, Life Technologies, S-008-5) M231 medium for 96h. Subsequently, the medium was exchanged for fresh 1% SMDS medium, and 10μM CCG-1423 (Tocris Bioscience, #5233) or the corresponding volume of DMSO (Sigma-Aldrich, #D5879) added directly the medium. Cells were then harvested after an additional 72h.

To depolymerize actin in human coronary artery endothelial cells, Latrunculin B (Lat B, 100nM, Calbiochem, #428020) or DMSO (Sigma-Aldrich, #D5879) was added at 96h after transducing with MRTF-B virus, and cells were harvested after additional 24h. Before adding the LatB, cells were also transferred to low-serum medium (2.5% FBS) for a 24h period.

To inhibit the YAP-TEAD interaction, 2μM verteporfin (Sigma-Aldrich, SML0534-5MG) or the corresponding volume of DMSO (Sigma-Aldrich, #D5879) was added to the medium after transduction with MRTF-B virus for 72h (human coronary artery SMCs). Cells were harvested for RNA extraction after an additional 24h.

### RT-qPCR

At the end of the culture period, cells were washed (PBS, P4417, Sigma-Aldrich), lysed (Qiazol, Qiagen, #79306), and RNA was isolated using the Qiagen miRNeasy mini kit (Qiagen, #217004) in a QIAcube workstation. The NanoDrop 2000c (Thermo Scientific) instrument was used to determine RNA purity and concentration. For quantification of specific transcripts, we used the StepOnePlus thermal cycler (Applied Biosystems), the QuantiFast SYBR Green RT-PCR kit (Qiagen, 204,156), and QuantiTect Primer assays (Qiagen) for *CHRM3* (QT00200354), *CHRM2* (QT00092134), *CSPG4* (QT00120407), *CAV1* (QT00012607), *SRF* (QT00084063), *ACTA2* (QT000088102), *18S* (QT00199367), *Chrm2* (QT00290297), *Chrm3* (QT00312774), *Srf* (QT00126378), and *18s* (QT02448075). The exact primer sequences are considered proprietary by Qiagen. As a house-keeping reference gene, we used *18S*, and the fold changes (null or vehicle) were calculated using the Pfaffl method. The relative transcript levels are represented by official gene symbol in italics in all graphs and the units are fold changes (FC) versus control.

### Correlation Analyses Using the GTExPortal.org

R-scripts and methods for downloading, and TMM normalizing, RNA-sequencing data from the GTExPortal.org have been described elsewhere (Krawczyk et al., 2015; Sward et al., 2019). For the current analyses, data were downloaded in the summer of 2020. Correlations between *MYOCD* and *CHRM3* were examined in transverse colon (*n=*406), urinary bladder (*n=*21), and terminal ileum (*n=*187) using the Spearman method in GraphPad Prism. Correlations between *MRTFB* and *CHRM3* were examined in the frontal cortex (*n=*209), tibial artery (*n=*663), aorta (*n=*432), and lung (*n=*578), respectively. Individual TPM (transcripts per million) values along with R- and P-values are given in the graphs or running text.

### Organ Culture and Treatment With CCG-1423

Six wild-type C57Bl/6 female mice, weighing 20–25g from ongoing breeding efforts, were euthanized by cervical dislocation. The abdomen was opened, and the urinary bladder was taken out and put in 2ml Eppendorf tubes prefilled with ice cold and sterile HEPES-buffered Krebs solution (135.5mm NaCl, 5.9mm KCl, 1.2mm MgCl2, 2.5mm CaCl2, 11.6mm glucose, 11.6mm HEPES, pH 7.4, and 0.5% PEST). A part of the stomach/ventricle was also excised, and the trachea and esophagus were removed together and put in prefilled tubes as above. The tissues were transported to the laboratory where the trachea and esophagus were dissected free from surrounding connective tissue, and the mucosa was removed from the urinary bladder and the ventricle under a dissection microscope. All tissues were cut into two equal pieces in the craniosacral direction. One piece was cultured with vehicle (DMSO) and the other with the MRTF-SRF inhibitor CCG-1423 (10μM) in DMEM Ham’s F12 medium with 50U/ml penicillin, 50μg/ml streptomycin, 2% dialyzed FCS, and 10nM insulin. Following organ culture for 96h, the tissues were frozen in liquid nitrogen. RNA was isolated using the RNeasy Minikit^®^ from Qiagen and mRNA levels were determined by RT-qPCR (StepOne^®^).

#### Wire Myography

The organ culture procedure was almost identical to that described above but using endothelial cell culture medium instead of DMEM/HAM F12. Caudal artery segments were cultured on myograph wires allowing for immediate mounting in a Mulvany myograph (610M; Danish Myo Technology) as described (Dahan et al., 2014) following culture. After stretching to a basal tension of 5 mN in the absence of Ca^2+^, and equilibration in normal Ca^2+^-containing HEPES buffer, arteries were depolarized using K^+^-high solution (60mM). Following washing 0.3μM cirazoline was added, and after 8min, carbachol (10–8 to 10–5M) was added in a cumulative manner. Average force (in mN) over the stimulation period was used for analysis.

### Protein Isolation and Western Blotting

After 120h of transduction with virus, cells were washed in ice cold PBS (Thermo Fisher Scientific, # 20012027) twice. They were harvested by scraping following addition of lysis buffer (70μl 60mM Tris-HCl, 2% SDS, 10% glycerol, pH 6.8). To prepare the reduced and unreduced samples in parallel, the lysates were adjusted to 1μg/μl with or without mercaptoethanol (5%) after determining the protein concentration (BIO-RAD DC protein assay kit, #500–0112). Lysates to which mercaptoethanol was added were also heated to 95°C for 5min. All the samples were stored at −80 °C. 25μg of protein was loaded per lane on AnyKd gels (BIO-RAD, #161–0395) along with PrecisionPlus Kaleidoscope markers (BIO-RAD, #161–0395). Gels were run at 200V until the front ran off using the Tris/Glycine/SDS buffer system (BIO-RAD, #161–0732). The Trans-Blot Turbo transfer system and 0.2μM nitrocellulose (BIO-RAD, #170–4159) were used for transfer. Following blocking for 2h in Casein block (BIO-RAD, #161–0782), membranes were incubated with CHRM3 primary antibody (Abcam, ab126168, 1:200) in sealed plastic bags. Bags were tumbled in the cold room (4°C) for 4days. Membranes were subsequently washed in Tris-buffered saline (BIO-RAD, 170–6435) with 0.1% Tween (BIO-RAD, 161–0781) three times (10min each), incubated with anti-rabbit HRP (horseradish peroxidase)-conjugated secondary antibodies (1:10000, Cell Signaling Technology, #7074S) for 2h, and washed again. The West Femto substrate (Thermo Fisher Scientific, #34096) and the Odyssey Fc Imager (LI-COR Biosciences) were used for detection. After initial detection, membranes were stripped in Stripping buffer (Thermo Scientific, 46430) for 30min, at 60°C, washed as above and blocked again for 2h in Casein block. Thereafter, membranes were incubated with HSP90 primary antibody (BD Biosciences, 610418, 1:1000) in the cold room (4°C) for 2days. After three washes as above, membranes were incubated with anti-mouse HRP (horseradish peroxidase)-conjugated secondary antibodies (1:10000, Cell Signaling Technology, #7076S) for 2h and washed again. Bands were normalized to HSP90 in the same lane.

### Inducible and SMC-Specific Knockout of Srf

B6.129S6-Srftm1Rmn/J mice were obtained from the Jackson laboratory (stock number #006658). These mice have loxP sites flanking promoter and exon 1 sequences of the Srf gene (Ramanan et al., 2005). The Srf-floxed mutant mice (Srf^fl/fl^) were bred with hemizygous Myh11-Cre/ERT2 mice (Wirth et al., 2008), allowing for knockout of Srf in smooth muscle upon treatment with tamoxifen (Daoud et al., 2021). Cre expression was induced by intraperitoneal injection of tamoxifen (1mg/mouse/day) in ethanol/sunflower oil (1:10) for 5 consecutive days. Floxed but Cre-negative mice treated with tamoxifen were used as controls in the first round. In the second round, we also included a group of floxed Cre-positive mice receiving vehicle as controls. Mice were killed by cervical dislocation and organs were excised and transferred to ice cold HEPES-buffered Krebs solution (135.5mmol/L NaCl, 5.9mmol/L KCl, 1.2mmol/L MgCl_2_, 11.6mmol/L HEPES, 11.5mmol/L glucose, and 143.8mmol/L Cl^−^, pH 7.35 at 37°C) with no Ca^2+^. After transportation to the laboratory, organs (urinary bladder, colon, ileum, trachea, esophagus, caudal artery, aorta, and kidney) were cleaned under dissection microscopes, quickly blotted on filter paper to remove excess solution, and frozen in liquid N_2_. After storage at −80 °C, RNA was isolated as described. Mouse primers for *Chrm3*, *Chrm2*, *Srf*, and *18s* were obtained from Qiagen. Primer sequences are considered proprietary information. Five wild-type and five knockout mice were used for the experiments with 10d induction, but two samples were lost to workup (one aorta and one bladder). Therefore, the n-value for these is only four. For 21d induction, we used 5 vehicle controls (VC), 5 tamoxifen controls (TC), and 12 tamoxifen knockouts (TKO), and no samples were lost to workup. There was no difference between the VC and TC groups in expression of *Srf* or *Chrm3* in ileum or aorta, but there was a borderline significant difference in the bladder (TC<VC). All differences in TKO bladder were highly significant versus both VC and TC and irrespective of data pooling (ANOVA-Tukey). We therefore pooled 21d control data (VC+TC) throughout for simplicity.

### Promoter Reporter Assay

The promoter reporter plasmid for *CHRM3* contained a dual-luciferase vector backbone and was from GeneCopoeia (HPRM30679). HEK293 cells were seeded in 24 well plates and transfection was conducted in antibiotic-free DMEM media with 10% fetal bovine serum (Thermo Fisher, #23320–002). The *CHRM3* plasmid was transfected together with either p3xFLAG-MKL1 plasmid (Addgene, #11978), p3xFLAG MKL2 plasmid (Addgene, #27175), or MYOCD plasmid (Origene, #SC327690,) using Lipofectamine 2000 (Thermo Fisher Scientific, #11668030). After 72h, the medium was collected, and the Secrete-Pair Dual Luminescence Assay Kit was used as recommended in the manufacturer’s protocol (GeneCopoeia, #LFO32). Signal was measured in a GloMax 20/20 Luminometer (Promega, #E5311) and the ratio of Gaussian luciferase and alkaline phosphatase (a proxy for transfected cell number) was taken as a measure of promotor activity.

### Single Cell RNA-Sequencing Data

To explore the possibility that different MRTFs dominate in different arterial cell types, we accessed a single cell RNA-seq dataset (He et al., 2018; Vanlandewijck et al., 2018) and extracted read count averages for different cell types. To plot the data, all read count averages for specific transcripts were normalized to the cell type with highest read count average of that transcript.

### Ca^2+^ Measurements

Cells grown on the glass-bottom dishes were transduced with MRTF-B or null virus for 120h and washed with HEPES-buffered Krebs solution (in mM: NaCl 135.5, KCl 5.9, MgCl_2_ 1.2, glucose 11.6, HEPES 11.6, and CaCl_2_ 2.5, pH 7.4) twice. Thereafter, cells were incubated with the intracellular calcium indicator Fluo-4, AM (5μM, Thermo Fisher Scientific, F14201) or X-Rhod-1, AM (1μM, Thermo Fisher Scientific, #X14210) and Pluronic F-127 (0.02% (w/v), Molecular Probes, #P-1572) in Krebs buffer at room temperature for 1h. Cells were again washed with buffer twice for 10min. Real-time Ca^2+^ imaging was done using a confocal microscope (LSM 5 PASCAL, Carl Zeiss, Germany). After 200s of data acquisition as a baseline, 3μM carbachol was mixed into the buffer, and fluorescence was recorded for another 200s. Thereafter, 1mM ATP was mixed into the buffer and acquisition was stopped after an additional 200s. The ZEISS ZEN microscope software was used to measure the fluorescence intensities in regions of interest (ROI). ROIs were positioned over the 20 cells with the largest relative response in all fields of view and the average intensity over these ROIs was used for statistical testing. F_0_ represents the mean of fluorescence intensity over the first 200s, and F_1_ represents the fluorescence intensity at any given time. Ca^2+^ changes were expressed as F_1_/F_0_ (%) and are plotted with 95% confidence intervals.

### Statistics

**Figure 3:**
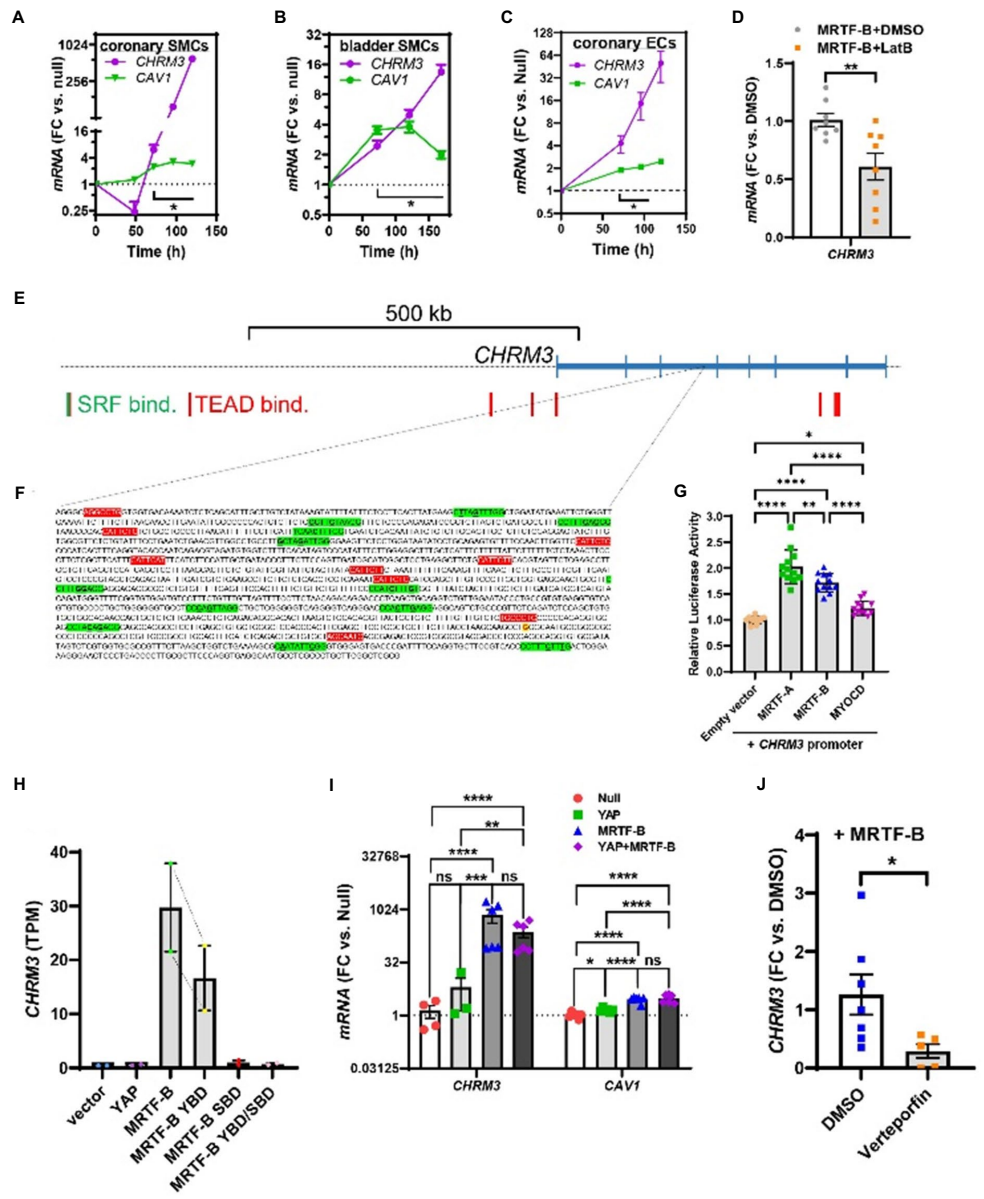
Time-course data, promoter reporter assays, and MRTF-B-YAP cooperation. MRTF-B was overexpressed in different cell types (200 MOI), and cells were harvested at different times. RNA was subsequently isolated, and *CHRM3* was measured by RT-qPCR. **(A)** Shows time-dependent upregulation of *CHRM3* in human coronary artery SMCs. Significant increases were seen at times exceeding 48h, and a 597-fold increase was seen at 120h (*p*=0.0004, *n=*3). Because there was no indication that the increase of *CHRM3* reached a plateau at longer transduction times, we designed an experiment using even longer incubations in human bladder SMCs (**B**). Again, there was no tendency of a plateau. Moreover, the maximal increase was somewhat smaller than in coronary artery SMCs. Similar results were obtained in human coronary artery endothelial cells [**(C)**, 200 MOI]. **(D)** Shows that *CHRM3* was reduced by Latrunculin B (100nM, gray bar) in MRTF-B-transduced ECs. Inspection of the *CHRM3* gene locus on chromosome 1 (**E**) revealed SRF binding (green vertical bars) and TEAD binding (red vertical bars) to many sequences 5' of, and over, the longest transcript (blue). Direct examination of a commercial promoter reporter sequence (NM_000740, transcript variant 2, FIGURE 3 | hg38; chr1+: 239627686–239,629,364; TSS=239,629,073) did not reveal any true CArGs, but 11 motifs with 2 deviations from the classical CArG sequence [CC(A/T)_6_GG, green highlights, deviations underlined, **(F)**] were present, along with 9 TEAD motifs [red highlights, **(F)**]. The transcription start site for the promoter is highlighted in yellow with red lettering. This “CArG-deficient” promoter responded to MRTFs in a luciferase reporter assay **(G)** run using HEK 293 cells. **(H)** Shows *CHRM3* mRNA expression in MCF10 cells transfected with YAP, MRTF-B, and two MRTF-B mutants; the YBD mutant does not bind YAP, and the SBD mutant does not bind SRF. **(I)** Shows the effects of YAP and MRTF-B transduction, alone and in combination, on *CHRM3* in human coronary artery SMCs. Ct values for *CHRM3* were sometimes too high for reliable detection (null and YAP). This is the reason why the sample size is less than *n=*6 for *CHRM3* in the null and YAP groups, even if six experiments were run for the panel. **(J)** Shows the effect of the YAP-TEAD inhibitor verteporfin in MRTF-B-transduced coronary artery SMCs. Two samples were lost in the verteporfin group again due to lack of amplification. ****p<0.0001, ***p<0.001, **p<0.01, and *p<0.05.

Statistical testing was done using log2-transformed expression data. For comparisons between two groups, we used the Mann-Whitney U test for unpaired data. In some panels, such as the time-course figures in Figures 3A-C, the controls used for statistical testing are not plotted in the graphs in the interest of clarity. Moreover, in Figures 3A-C, the sample size was too small for Mann-Whitney testing, and we therefore used student t-test. For multiple comparisons, one-way ANOVAs followed by Tukey’s post-hoc test was used. The residual distributions (QQ plots) of the log2-transformed RT-qPCR data in the ANOVAs were linear with a slope of 1, supporting a normal distribution. Two-way ANOVAs were used in the Ca^2+^ imaging experiments.

## Results

### RNA-Sequencing Shows That *CHRM3* is Regulated by Myocardin

We first generated an RNA-sequencing dataset for identification of transcripts regulated by myocardin. Myocardin was overexpressed using an adenoviral vector (Ad-CMV-MYOCD) in cultured human coronary artery SMCs. With 36–49 million pair end reads per sample and four samples per group, this dataset provides good transcriptome coverage for several downstream applications. Among the differentially expressed transcripts (the differential expression analysis is provided in the supplementary data), we noted that the muscarinic M_2_ receptor (*CHRM2*), the M_3_ receptor (*CHRM3*), and the M_5_ receptor (*CHRM5*) were increased 8days after overexpression of myocardin compared to null adenovirus (Figure 1B, brackets give adjusted P-values). For independent confirmation, we assayed *CHRM3* alongside a positive control (*CSPG4* or *CAV1;* Krawczyk et al., 2015; Rippe et al., 2021) using RT-qPCR at four days of transduction with Ad-CMV-MYOCD or Ad-CMV-null viruses. *CHRM5* was not examined further due to uncertainty regarding its biological function, and *CHRM2* was not detectable with the primer assay used, but the increase of *CHRM3* was readily confirmed (Figure 1C). We also measured *CHRM3* in cultured human bladder SMCs and again observed an increase following transduction of myocardin (Figure 1D). We concluded that overexpression of myocardin increases the transcript level of the muscarinic M_3_ receptor in different human SMCs.

### All MRTFs Increase *CHRM3*

We have previously reported that myocardin correlates with some of its target genes at the mRNA level (Krawczyk et al., 2015; Sward et al., 2019). To examine if this was the case for *CHRM3*, we used human RNA-seq data downloaded from the GTExPortal.org (Consortium, 2013). Correlations were examined in different organs using the Spearman method. *MYOCD* correlated tightly with *CHRM3* in the transverse colon (Figure 2A), the urinary bladder (Figure 2B), and the ileum (Figure 2C). *MYOCD* also correlated with *CHRM3* in the coronary artery (*R=*0.46, *p*<0.0001, not shown), but not in the other two arteries represented in the database (aorta and tibial artery, not shown). Similarly, *MYOCD* did not correlate with *CHRM3* in the brain (frontal cortex, not shown). We therefore instead tested if *MRTFA* and *MRTFB* correlate with *CHRM3*, and we observed a strong positive correlation for *MRTFB* in the brain (Figure 2D). This was also seen in the lung, where *MRTFB* correlated with *CHRM3* (Figure 2E), in the tibial artery (*MRTFB* vs. *CHRM3*: *R=*0.32, *p*<0.0001, not shown), and the aorta (*MRTFB* vs. *CHRM3*: *R=*0.28, *p*<0.0001, not shown). These analyses suggested that MRTF-B may increase *CHRM3* like *MYOCD* and therefore prompted us to experimentally determine if all MRTF family members increase *CHRM3* expression. Indeed, in side-by-side adenoviral transductions, all MRTFs increased *CHRM3* (Figure 2F), and the effect of MRTF-B was larger (34-fold) than the effects of MYOCD (5-fold) and MRTF-A (5-fold) at the same virus titers. This difference between MRTFs is likely real, because MRTF-B did not increase *CAV1* more effectively than MYOCD in the same samples (Figure 2G). All MRTFs thus have the capacity to regulate transcription of the muscarinic M_3_ receptor in human SMCs, but MRTF-B appears most effective in this regard.

### The MRTF-SRF Inhibitor CCG-1423 Reduces *CHRM3* in MRTF-Transduced Cells

From a therapeutic point of view, it is important to examine if substances that have been developed to inhibit MRTF-SRF signaling (Bell et al., 2013; Lundquist et al., 2014) also affect expression of the muscarinic M_3_ receptor. One of these substances is CCG-1423, and it inhibits serum response element-driven gene activation with an IC_50_ value of 1–5μM via interference with MRTF-SRF-dependent transcriptional activation (Evelyn et al., 2007). Indeed, CCG-1423 (10μM) reduced *CHRM3* at 72h of treatment in MRTF-transduced SMCs (Figure 2H). The effect appeared greater in cells transduced with MRTF-B than in cells transduced with MRTF-A, which may reflect the larger effect of MRTF-B on *CHRM3*. No effect was seen without MRTF transduction (not shown), or at earlier times (not shown), findings that we attribute to low basal M_3_ levels in cultured SMCs.

### Short Hairpin Silencing of SRF Reduces *CHRM3* Expression

To examine the SRF-dependence of the MRTF effect on *CHRM3*, we next used a short hairpin construct (Ad-shSRF, two virus titers) to knock down SRF in cells transduced with MRTF-A. The levels of *SRF*, *ACTA2*, and *CHRM3* were determined by RT-qPCR in silenced and control cells (U6). We found that *CHRM3* was reduced upon SRF silencing (Figure 2I, green), paralleling the established target gene *ACTA2* (Figure 2I, black/pink triangles). The MRTF-A effect on *CHRM3* thus requires SRF.

### Time-Course Studies

To better estimate the full effect-size of MRTF-B, we next performed time-course studies where we harvested human coronary artery SMCs at various times after coactivator transduction. The effect on *CHRM3* was compared with the effect on *CAV1* (Krawczyk et al., 2015). After an initial small drop, *CHRM3* started to increase at 72h after MRTF-B transduction (Figure 3A), and it continued to increase beyond 96h without any sign of saturation. At 120h, *CHRM3* was increased 597-fold (*p=*0.0004), and this effect dwarfed the effect on *CAV1* in the same samples. Of note, 72h was required for increases of both *CAV1* and *CHRM3* to become significant. We next designed a similar experiment for bladder SMCs, but with even longer incubations to hopefully capture the full range of regulation. Again, *CHRM3* increased well beyond 100h while *CAV1* did not (Figure 3B) and there was no sign of saturation. We also tested the effect of MRTF-B in human coronary artery endothelial cells (ECs) but failed to saturate the effect (Figure 3C). While significant, the effect on *CHRM3* in both ECs and bladder SMCs appeared somewhat smaller than in coronary SMCs (compare Figures 3A–C). However, Ct values for *CHRM3* in coronary artery SMCs were high to start with (Ct rank order for *CHRM3*: bladder SMCs<coronary SMCs<ECs), suggesting a low basal level of expression compared to bladder SMCs. In keeping with the sensitivity of MRTFs to actin dynamics, we found that depolymerization of actin (Latrunculin B: LatB, 24h) in MRTF-B transduced ECs reduced *CHRM3* (Figure 3D).

### Promoter Reporter Data and Dependence on YAP-TEAD

Human chromatin immunoprecipitation sequencing data (Encode3, UCSC genome browser) did not reveal SRF binding in the immediate vicinity of *CHRM3* on chromosome 1, even if SRF binding was noted >500kb upstream of the gene (Figure 3E, green vertical bar, far left). Binding of TEA domain transcription factors (TEADs), known to be important for YAP/TAZ and a subset of MRTF-controlled genes (Kim et al., 2017), was seen over and near the *CHRM3* locus (Figure 3E, red vertical bars). We also inspected CArG box predictions in the mouse that are conserved in man and found 1 CArG box ≈18.5kb upstream of *Chrm3* (on chromosome 13, not shown), and an additional 10 conserved CArG boxes evenly distributed across the *Chrm3* locus (not shown). Finally, manual inspection of a commercial promoter reporter sequence revealed lack of perfect CArGs motifs, but 12 motifs with two deviations each from the canonical CArG sequence (Figure 3F, green highlights, deviations underlined). This promoter also contained nine TEAD motifs (so called MCAT motifs, red highlights in Figure 3F), six of which are predicted to be functional based on identical motifs in other promoters. Co-transfection of this reporter with MRTFs caused 2-fold activation (HEK 293 cells, Figure 3G), albeit not with the natural rank order of efficacy. Taken together, these analyses suggest that there are many conserved CArGs (11) that could contribute to regulation distributed over the *CHRM3* locus and that a *CHRM3* promoter that lacks true CArGs is activated by MRTFs.

Previous work demonstrated that both MRTF-A and MRTF-B may bind YAP-TEAD to activate a reporter with 8 TEAD-binding motifs and no SRF-binding motifs (Kim et al., 2017). The same study also demonstrated MRTF-B shows a preference for binding to YAP-TEAD, whereas MRTF-A shows a preference for binding to SRF. The stronger effect of MRTF-B compared to MRTF-A and MYOCD on *CHRM3* may therefore depend on dual activation of YAP-TEAD and SRF-dependent transcription. To approach this possibility, we interrogated an RNA-sequencing dataset with two replicates, where MRTF-B was overexpressed alongside YAP-binding deficient (YBD) and SRF-binding deficient (SBD) mutants of MRTF-B, as well as a double mutant (YBD/SBD). In this dataset (Kim et al., 2017), *CHRM3* was increased by MRTF-B compared to empty vector as shown in Figure 3H. The effect of the YBD mutant was 45% smaller than control MRTF-B, and the SBD mutant was without effect (Figure 3H). This independently supports a key role of SRF, but also bolsters the idea that MRTF-B may depend on YAP-TEAD. For such target genes, remarkable synergy was reported on combined overexpression of MRTF-B and YAP compared to overexpression of MRTF-B or YAP alone (Kim et al., 2017). Therefore, we next examined the possibility that YAP and MRTF-B act in synergy. This was done by overexpressing these coactivators alone and together. However, overexpression of YAP alone had no significant effect, and it did not boost the effect of MRTF-B (Figure 3I). Overexpressed YAP had a transcriptional impact, because it increased *CAV1* in the same cells, albeit not as effectively as MRTF-B (Figure 3I).

To further probe if YAP-TEAD signaling plays a role for *CHRM3* expression, we used an inhibitor. Verteporfin was identified in a screen for inhibitors of the YAP-TEAD interaction (Liu-Chittenden et al., 2012), and it has been used in numerous reports to study the functional role of this transcriptional complex. Here, cells were transduced with MRTF-B for 3 days, and 2μM verteporfin or vehicle was added for an additional 24h. Verteporfin reduced the *CHRM3* transcript compared to vehicle (Figure 3J). The size of this effect is underestimated by the data in Figure 3J, because two samples were lost in the verteporfin group on account of insufficient amplification. Taken together, these findings further suggest that YAP-TEAD signaling is necessary but not sufficient for *CHRM3* expression and that YAP-TEAD and MRTF-SRF likely act cooperatively to drive *CHRM3* expression.

### Detection of the CHRM3 Protein in MRTF-B Transduced Cells by Western Blotting

**Figure 4:**
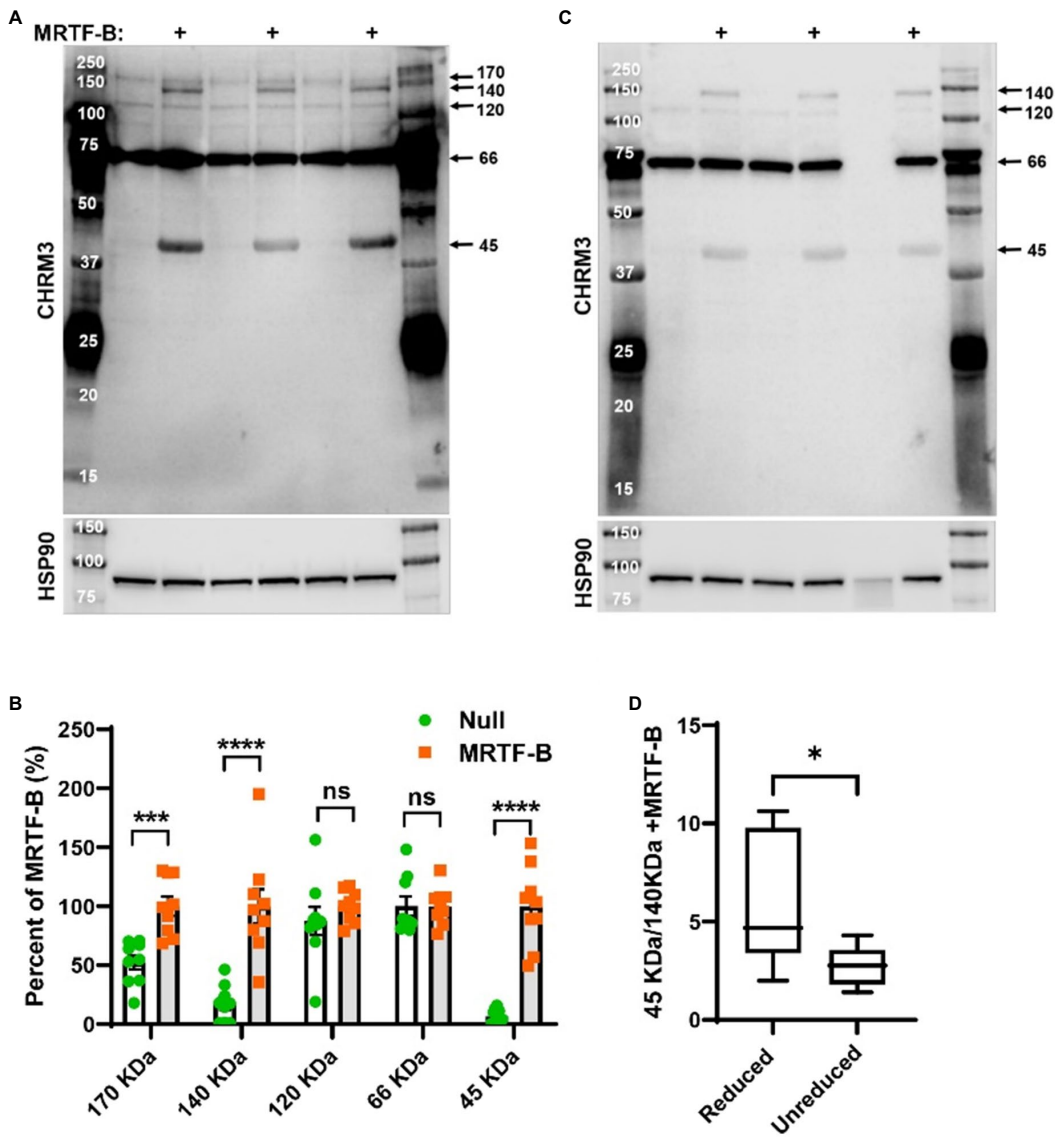
MRTF-B increases CHRM3 immunoreactive bands in human coronary artery smooth muscle cells. Protein lysates from human coronary artery SMCs treated with null virus and with MRTF-B virus for 120h, respectively, were used for Western blotting using an antibody raised against a human CHRM3 in **(A)**. After developing the blot, it was stripped and incubated with HSP90 antibody (shown below) to assess equal protein loading. At least five bands were detected with the CHRM3 antibody, and three bands at ≈170, ≈140, and ≈45kDa changed significantly with MRTF-B as shown in the compiled analysis in (B). Quantification in panel **(B)** was done using the blots in **(A)**. Proteins from the same original lysates were also prepared in non-reducing conditions, to examine if the relationship between bands changed **(C)**. The volume was insufficient for one of the null samples in this experiment (lane 6). Non-reducing conditions favored the 140kDa band at the expense of the 45kDa band **(D)**, suggesting multimerization. ****p<0.0001, ***p<0.001, and *p<0.05.

Previous work on a modified and tagged version of rat *Chrm3* revealed that M_3_ monomers migrate at 45kDa, dimers at 90kDa, and multimers at >120kDa, along with a proteolytically processed dimer at 75kDa (Zeng and Wess, 1999). Using an antibody raised against a peptide from human CHRM3, we observed three bands at ≈45, ≈140, and ≈170kDa that increased in MRTF-B transduced cells, with the most prominent changes occurring at ≈45kDa, and at ≈140kDa (Figures 4A,B). The antibody also detected bands at 66 and 120kDa, but the latter did not change in MRTF-B transduced cells (Figures 4A,B), and the 66kDa band, which was the strongest, was also seen in the lanes with molecular weight markers (Figure 4A). Because disulfide bridge-dependent multimerization (Zeng and Wess, 1999) was found to be responsible for Chrm3 bands at higher molecular weights, we next prepared the same protein lysates without reducing agent and boiling (Figure 4C). Insufficient lysate was available for one of the samples (Figure 4C, lane 6). Careful quantification showed that the 45kDa band declined at the expense of an increase of the 140kDa band in non-reducing conditions compared to reducing conditions (Figures 4C,D). The 45kDa and 140kDa bands are therefore interdependent species. Taken together, these findings support MRTF-B-driven increases of protein bands, likely monomers and trimers, that interact with an antibody against human CHRM3.

### CCG-1423 Reduces *Chrm3* in Organ Cultured Esophagus

**Figure 5:**
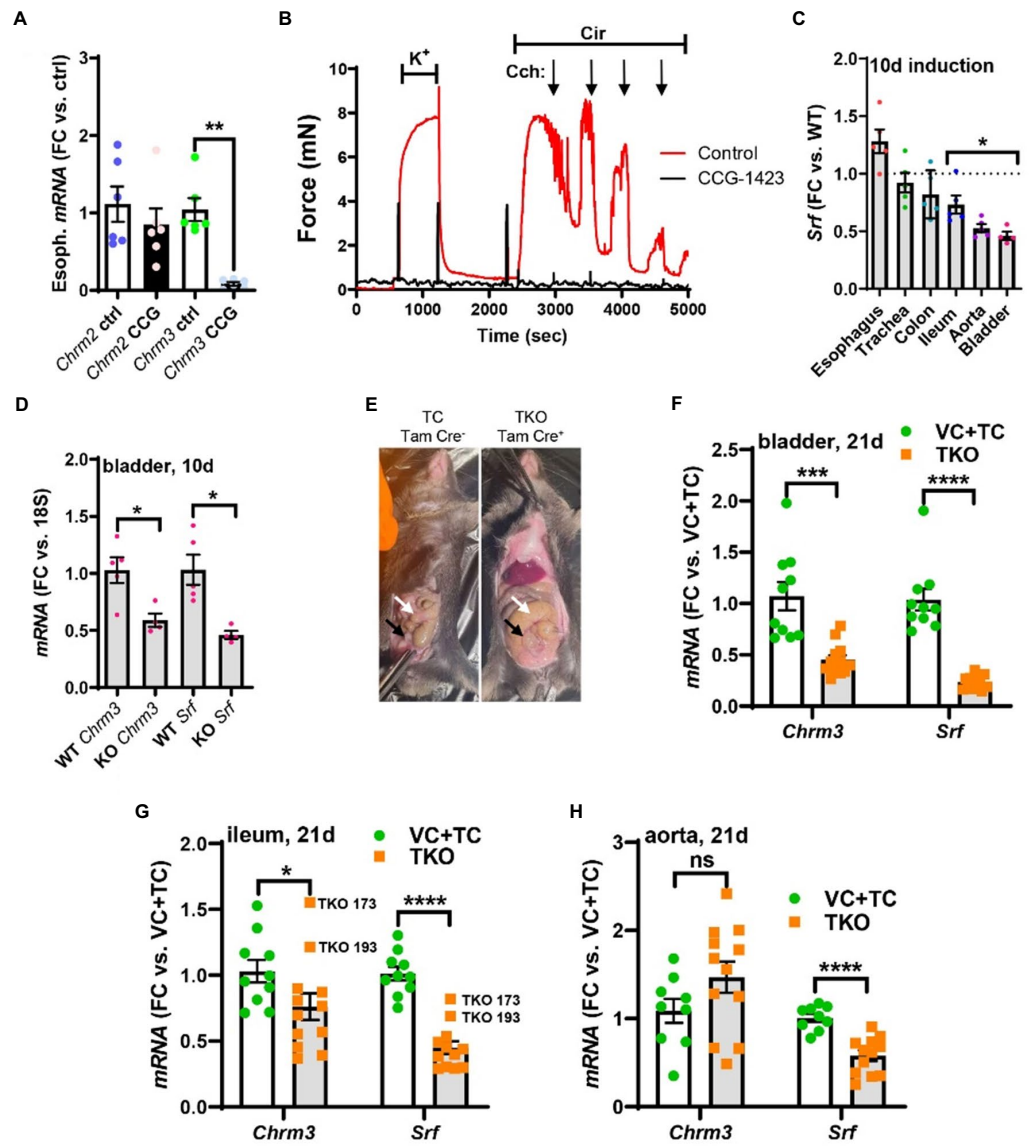
Pharmacological inhibition of MRTF-SRF signaling in organ culture, and knockout of Srf *in vivo*, reduces M_3_ receptor expression. To examine if MRTF-SRF signaling regulates muscarinic M_3_ receptor expression *in situ*, we first isolated organs from wild-type C57Bl/6 mice. Organs were split in half and maintained for 96h in organ culture with vehicle (DMSO) or CCG-1423 (10μM). A clear reduction of *Chrm3* relative to the house-keeping gene *18s* was seen in the esophagus **(A)**, but in the remainder of the organs, the house-keeping genes examined declined (not shown). We also found that organ culture of the mouse caudal artery in the presence of CCG-1423 eliminated force development on stimulation with the α1-adrenergic agonist cirazoline (**B**), suggesting that this experimental paradigm is unsuitable for studying effects on endothelium-dependent dilatation. Mice with SMC-specific knockout of Srf were next obtained by injecting Srf^fl/fl^ mice harboring the Myh11-Cre/ERT2 transgene with tamoxifen for 5 consecutive days (knockout: KO). Cre-negative Srf^fl/fl^ mice injected with tamoxifen were used as controls (wild-type: WT). Organs were harvested and frozen 10days after the first injection and transcript levels were determined by RT-qPCR. At this time, body weights were unchanged, but Srf depletion was seen in some organs **(C)**. **(D)** Shows that *Chrm3* was reduced in parallel with *Srf* in the bladder, but this was not seen elsewhere (not shown). We therefore next used mice at 21days post tamoxifen. Two control groups were included in this second experiment along with the tamoxifen-treated knockouts (TKO): vehicle-treated Cre-positive mice (VC) and tamoxifen-treated Cre-negative mice (TC). At 21days, mobility on provocation was reduced, the intestines had started to swell [**(E)**, white arrows], and the urinary bladders were often enlarged [**(E)**, black arrows]. Both *Chrm3* and *Srf* were reduced in the bladder **(F)** and ileum **(G)**. For the ileum, the two knockouts with the most modest *Srf* depletion (TKO 173 and TKO 193) are highlighted. No change of *Chrm3* was seen in the aorta **(H)**, despite significant *Srf* depletion. These findings show that MRTF-SRF signaling is critical for *Chrm3* expression in gastrointestinal and urogenital organs *in vivo*. ***p<0.001, **p<0.01, and *p<0.05.

Our loss of function experiments so far depended on prior overexpression of MRTFs in cell culture. To bypass the need for MRTF overexpression, we isolated organs from C57Bl/6 mice and maintained them in organ culture for 96h with and without CCG-1423 (10μM). Organs were harvested and frozen at the end of the culture period, and RNA was extracted. Three out of four of the organs (trachea, stomach, and bladder) did not cope well with organ culture with CCG-1423 for 96h, showing sizeable reductions of the house-keeping genes examined (*18s*, *Gapdh*), but in the esophagus, *Chrm3* was reduced with CCG-1423 (Figure 5A), and the decline of *18s* was small. *Chrm2* levels remained unchanged, but variability was considerable. This supported the view that *Chrm3* may be controlled by MRTFs in mouse cells *in situ*.

We also measured force in wire myographs after organ culture with CCG-1423. Both force development in response to the α1-adrenergic agonist cirazoline (Cir, 0.3μM), and relaxation in response to the muscarinic agonist carbachol (Cch, 10–8 to 10–5M), were maintained after 96h using the mouse caudal artery (endothelial cell culture medium; Figure 5B, red tracing), but inclusion of CCG-1423 during culture essentially eliminated force (Figure 5B, black tracing, *n=*6, *p*<0.001). Attempts to knock down *Srf* in organ culture, using the short hairpin used in human cells above, were also not successful (not shown). This called for a more robust and specific method to manipulate MRTF-SRF signaling in intact organs.

### Inducible and Smooth Muscle-Specific Deletion of Srf *in vivo*

To examine regulation of *Chrm3* by MRTF-SRF signaling *in vivo*, we generated smooth muscle-specific and inducible knockouts (KOs) of Srf (Park et al., 2015). Any changes in whole tissue lysates in this model should reflect changes in smooth muscle. To generate KOs, we treated homozygous Srf-floxed mice harboring a tamoxifen-regulated and smooth muscle-specific (*Myh11* promoter driven) Cre transgene with tamoxifen. Cre-negative floxed mice treated with tamoxifen were used as controls (WT). Srf knockout in SMCs results in intestinal pseudo-obstruction starting 21days after the first injection (Park et al., 2015) due to reduced cholinergic SMC contraction and impaired gastrointestinal motility (Angstenberger et al., 2007; Mericskay et al., 2007; Park et al., 2015). Here, organs were initially isolated for RT-qPCR on day 10 after the first of five injections. We picked this time based on our previous observation that knockout of YAP and TAZ, using the same Cre-deleter mouse and injection protocol, causes a ≈75% reduction in colon at 10days (Daoud et al., 2021). No evidence of animal discomfort, such as ruffled fur, reduced mobility, or kyphosis, was observed here at 10days, and body weights remained unchanged (not shown). 10days therefore represent the pre-symptomatic stage. Various organs were isolated, and *Srf* levels were determined by RT-qPCR. *Srf* was depleted by 54±4% in the urinary bladder, 47±3% in the aorta, and 26±8% in the ileum (Figure 5C), but no significant reductions were seen in the remainder of the organs examined. *Chrm3*, but not *Chrm2* (not shown), levels were reduced in the urinary bladder from KO compared to WT mice (Figure 5D). Reduction of *Chrm3* was not significant elsewhere (not shown), but *Chrm3* reduction correlated with *Srf* depletion across all organs (*p=*0.044, *R=* 0.44, Pearson, not shown).

In view of the rather limited changes at 10days, we next tried a longer induction time (21days). This time we used two control groups. One group of mice were Cre-postive and treated with vehicle (VC: vehicle control). Another group of mice were Cre-negative, and they received tamoxifen (TC: tamoxifen control). The third group included mice that were Cre-positive and that were treated with tamoxifen (TKO: tamoxifen treated knockouts). The two control groups were not different and were therefore pooled in the final analysis. At 21d, mobility on provocation was reduced in several knockout animals, and intestinal swelling was apparent in most of them (Figure 5E). The mice appeared healthy in most other regards. 21d therefore represents the early clinical phase. Reductions of *Srf* and *Chrm3* in the urinary bladder were augmented at 21d compared to 10d (compare Figures 5F,D). *Chrm3* was now also reduced in the ileum (Figure 5G) but not in the aorta (Figure 5H). Interestingly, two ileum samples (TKO 173 and TKO 193) with poor *Srf* depletion also had poor *Chrm3* depletion (Figure 5G). Taken together, these findings show that *Chrm3* in the bladder is reduced already in the pre-symptomatic phase and that the early clinical phase coincides with depletion of intestinal *Chrm3* in SMC-specific Srf knockouts. Maintained *Chrm3* expression in the aorta may be due either to a threshold effect, because Srf was less forcefully reduced, or to preferential expression in non-SMCs, such as endothelial cells. We favor the latter explanation for reasons given below.

### MRTF-B Increases the Responsiveness to the Muscarinic Agonist Carbachol in SMCs

**Figure 6:**
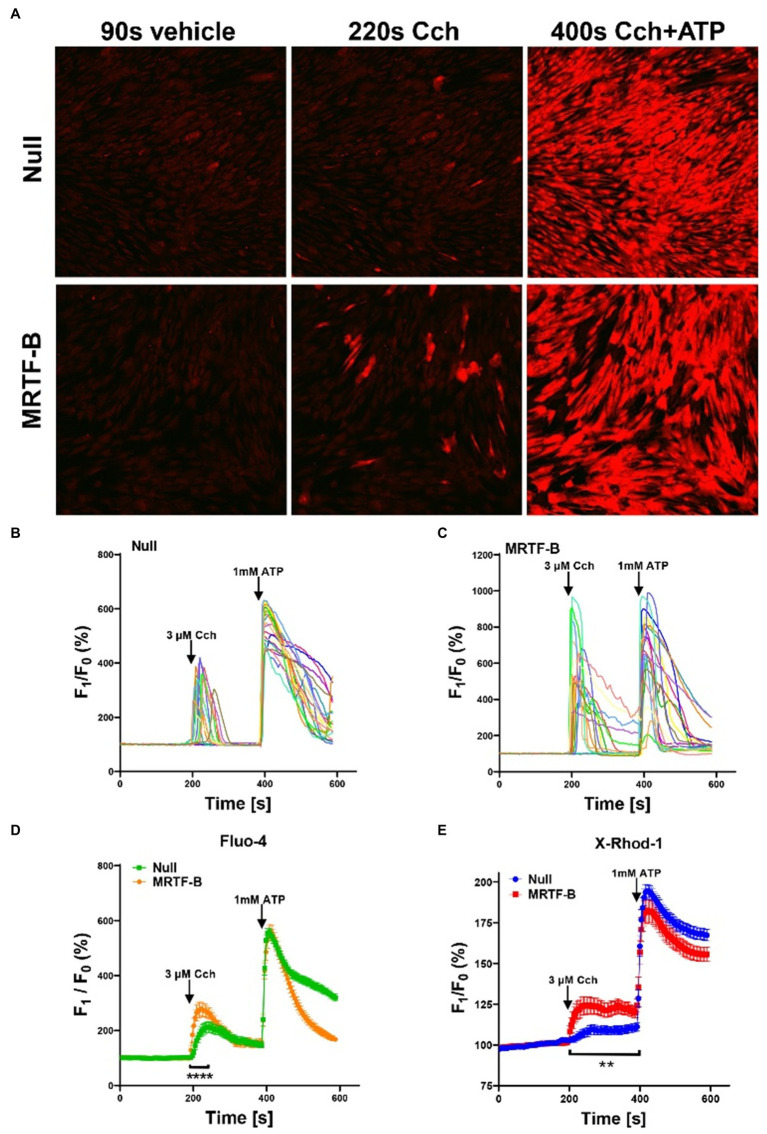
Ca^2+^ imaging of MRTF-B-transduced human coronary artery SMCs. To examine if MRTF-B transduction increases responsiveness to the muscarinic agonist carbachol, cells were treated as indicated for 5 days in culture. They were subsequently washed and loaded with Fluo-4 and imaged using confocal microscopy. Only a fraction of the cells responded to carbachol [**(A)**, middle], but the responses were larger in MRTF-B-transduced cells. **(B**,**C)** Show the 20 cells responding best to carbachol in the experiment in (A). (D) shows compiled data from three independent experiments with Fluo-4. The Ca^2+^ signal between 200 and 250s was significantly increased by prior MRTF-B transduction. (E) shows intracellular Ca^2+^ in human coronary artery SMCs after transduction of MRTF-B or null virus, respectively (*N* =6), but measured Ca^2+^ using X-Rhod-1. Error bars in **(D**,**E)** represent 95% confidence intervals. ****p<0.0001 and **p<0.01.

To support the concept that MRTF-SRF signaling regulates cholinergic responsiveness, human coronary artery SMCs that had been transduced with MRTF-B, were loaded with the fluorescent indicator Fluo-4, and stimulated with the muscarinic agonist carbachol (3μM) and ATP (1mM). Many cells were unresponsive to carbachol (Figure 6A, middle), both in the control group, and after MRTF-B transduction. This may relate to a low transduction efficiency (30–50% of all cells) and the short transduction time. However, MRTF-B transduced cells responded more forcefully to carbachol (Figures 6B,C), and data from three independent experiments showed that the peak response (200–250s) to carbachol was significantly increased in the MRTF-B group compared to the control group (Figure 6D).

With the intention of measuring Ca^2+^ only in transduced cells, we also used X-Rhod-1 for Ca^2+^ measurements. X-Rhod-1 reports both cytosolic and mitochondrial Ca^2+^, but it fluoresces at a wavelength compatible with the tagged MYOCD that was available to us. After pilot experiments, we did not follow through with the MYOCD experiments, but overexpression of untagged MRTF-B again enhanced the Ca^2+^ response to carbachol, while the Ca^2+^ response to ATP was similar (Figure 6E). In our loading conditions, only a minority of the cells (≈8%) showed a mitochondrial staining pattern, and in most cells, staining appeared to be diffusely cytoplasmic (not shown). Taken together, use of two different fluorescent Ca^2+^ indicators therefore support the view that MRTF-B promotes cholinergic responsiveness in cultured human SMCs.

### MRTF-B is the Dominating MRTF in ECs

**Figure 7:**
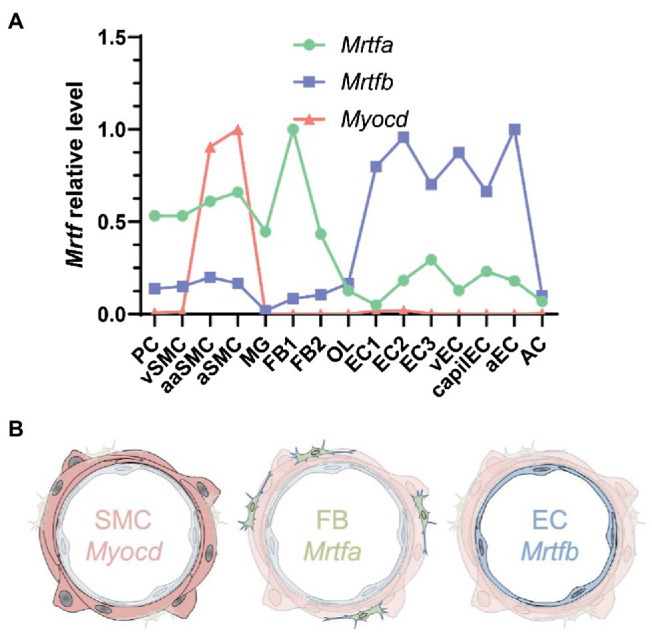
MRTF distribution across the vascular wall. **(A)** Shows cell-averaged mRNA expression data for the indicated transcripts in different cerebrovascular cell types. PC, pericytes; SMC, smooth muscle cells; MG, microglia; OL, oligodendrocytes; FB, fibroblasts; EC, endothelial cells; AC, astrocytes; v, venous; cap, capillary; a, arterial; aa, arteriolar; and 1, 2, and 3, subtype. **(B)** Shows a schematic representation of Myocd in SMCs, Mrtfa in FBs, and Mrtfb in ECs.

To better understand MRTF expression in different vascular cell types, we next examined a single cell RNA-sequencing dataset for cerebrovascular cells (Vanlandewijck et al., 2018). *Myocd* was enriched in arteriolar and arterial SMCs as expected (Figures 7A,B, pink). *Mrtfa* was highest in fibroblasts, but sizeable expression was also seen in pericytes and SMCs (Figures 7A,B, green). *Mrtfb* was enriched in endothelial cells (ECs, Figures 7A,B, blue). This provided a possible explanation for the lack of effect of SMC-specific Srf knockout on *Chrm3* in lysates of the whole aorta.

## Discussion

The research effort described here aimed to test the hypothesis that the muscarinic M_3_ receptor (*CHRM3*) is regulated by MRTF-SRF signaling using a combination of gain and loss of function approaches *in vitro* and *in vivo*. We demonstrate that the muscarinic M_3_ receptor transcript (*CHRM3*) is increased after overexpression of MRTFs, well known for their ability to respond to mechanical forces and actin dynamics (Olson and Nordheim, 2010). *CHRM3* was moreover reduced by SRF depletion in cultured SMCs and in the intact urinary bladder and ileum. Our findings suggest that M_3_ in endothelial cells is regulated by MRTF-B-SRF, while in SMCs, the combined influence of all MRTFs (*MYOCD*>*MRTFA*>*MRTB*) may be relevant. We demonstrate that MRTF-B is a more effective transactivator of M_3_ than MYOCD when both are overexpressed in the same cell type in parallel. This difference may allow for tissue-specific targeting of receptor expression should more selective substances to inhibit MRTF-SRF signaling be developed. This would be important since current drugs, such as the muscarinic antagonists, have serious side effects (e.g., dryness of mouth) that limit their clinical utility.

Our experiments show that the effect of MRTFs on *CHRM3* is difficult to saturate. This differs from the effect on *CAV1* which readily saturates. We are uncertain of the reason for this, but we note that multiple rather poor CArG-like sequences and numerus TEAD motifs are present in one of the promoters (and indeed across the entire gene locus). We also find that this promoter is activated in a luciferase reporter assay. One possibility, therefore, is that binding between MRTFs and the promoter involves (1) multiple weak SRF interactions, that are difficult to detect by ChIP-seq, in addition to (2) direct binding to YAP-TEAD (Kim et al., 2017), such that many productive complexes form at high concentration of MRTFs. If true, this would imply that cells need to proceed relatively far toward the SMC fate for M_3_ to be expressed at a meaningful level and that both YAP-TEAD and MRTF-SRF signaling are needed. Such a model fits available pharmacological and knockout data [this study, (Daoud et al., 2021)] and could perhaps explain differences in M_3_ expression between different types of SMCs (i.e., gastrointestinal vs. vascular). However, differences in the MRTF-B/MYOCD ratio are an equally plausible explanation for differences in M_3_ expression between cell types.

It is now four decades ago that endothelium- and nitric oxide-dependent dilatation of arteries by acetylcholine was discovered (Furchgott and Zawadzki, 1980; Palmer et al., 1987). It remained unclear for many years what the physiological role of this phenomenon is, but meticulous work conducted over the last decade has established autocrine cholinergic signaling in endothelial cells as critical for flow-mediated dilatation (Wilson et al., 2016). Moreover, it is only recently that the transcriptional control mechanisms responsible for endothelium-dependent dilatation have started to be uncovered. Several groups, including ours, have demonstrated that soluble guanylyl cyclase, the major nitric oxide receptor in the vascular media, is regulated by NOTCH signaling (Chang et al., 2011; Rippe et al., 2017). It has also been demonstrated that Srf in SMCs is important for nitric oxide-dependent dilatation (Galmiche et al., 2013), but the precise mechanism of that effect remains to be identified. One possibility is that it occurs via *Kcnmb1*, an important subunit of the large conductance Ca^2+^-activated potassium channel, which is regulated by Myocd-Srf (Long et al., 2009). This ion channel plays a key role in nitric oxide-dependent dilatation (Leo et al., 2014). However, additional effectors cannot be ruled out (Galmiche et al., 2013). By implicating MRTF-B-SRF in control of muscarinic M_3_ receptors in endothelial cells, our current findings add another layer of regulation to this complex mode of communication between cell types in the vascular wall.

Our experiments on Srf-deficient mouse tissues show reduction of M_3_ receptor transcripts in the urinary bladder and ileum *in vivo* at 21days. It is interesting to note that the early clinical phase of this model coincides with intestinal depletion of *Chrm3*. No change of *Chrm3* was seen in the aorta, and we suspect that maintained *Chrm3* expression in the aorta is due to preserved MRTF-B-SRF signaling in the endothelium. An endothelial-specific Srf knockout will be needed to address this hypothesis, or, alternatively, dual knockout of MRTF-A and MRTF-B in the endothelium. Similarly, myocardin knockout in SMCs would be required to prove a role for myocardin in *Chrm3* expression in this cell type. Thus, specific deletion of MRTF alleles in different vascular cell types would have further strengthened our conclusions, but this was considered beyond the scope of the present work. SMC-specific and inducible Srf knockout, as was done here, eventually result in death from intestinal pseudo-obstruction, associated with reduced gastrointestinal transit *in vivo* and reduced cholinergic activation of colon and bladder preparations *in vitro* (Angstenberger et al., 2007; Mericskay et al., 2007; Park et al., 2015). Given that colonic motility and cholinergic contractility are similarly reduced in M_3_-deficient mice (Kondo et al., 2011), it seems plausible that depletion of M_3_ receptors represents one molecular mechanism for reduced contraction in Srf knockouts. Indeed, mutations in *CHRM3* in humans cause “prune belly syndrome” with combined intestinal and bladder distension (Weber et al., 2011).

Bioinformatics analyses demonstrate correlations between *MYOCD* and *CHRM3* in human gastrointestinal and urogenital organs. This reinforces the view that MYOCD-SRF is an important, perhaps major, transcriptional control mechanism for *CHRM3* in these organs, but it does not rule out other control mechanisms. One such mechanism likely involves YAP-TEAD as suggested by our previous studies on inducible YAP/TAZ knockout mice (Daoud et al., 2021), and by pharmacological data herein. In the brain and lung, and in some arteries, *MYOCD* did not correlate with *CHRM3*. Instead, we observed correlations with *MRTFB*. In arteries, we believe that these correlations are driven primarily by the regulation of *CHRM3* by MRTF-B in endothelial cells because we found that MRTF-B increased the level of *CHRM3* in this cell type in culture. We did not examine if MRTF-B regulates *CHRM3* in neurons. However, it has been demonstrated that Mrtf-a and Mrtf-b together play essential roles for brain development (Mokalled et al., 2010). Perhaps, these MRTFs remain important for neuronal gene expression in adult life, contributing to the correlations observed in brain.

The activities of MRTF-A and MRTF-B depend on dynamics of the actin cytoskeleton (Olson and Nordheim, 2010). This implies that intracellular effectors of actin filament formation, such as Rho-associated kinase, may influence M_3_ expression. We found that Latrunculin B, which depolymerizes actin, reduces M_3_ in cells transduced with MRTF-B. Prior work demonstrated that Rho-associated kinase, which polymerizes actin, reduces eNOS expression (Ming et al., 2002). There may thus be a balancing influence of increased actin filament formation on M_3_ (predicted to increase) and eNOS (predicted to fall), which could leave cholinergic dilatation unchanged, but this remains to be examined. Given the reported activation of MRTF-A by substrate stiffness (Jain et al., 2013; Foster et al., 2017), it may be considered that M_3_ levels increase in, e.g., fibrotic disease. Matrix stiffness could perhaps also contribute to the paradoxical vasoconstriction of arteries in response to acetylcholine that is seen in certain pathological conditions (Ludmer et al., 1986), but it could also be that endothelial damage simply uncovers direct activation of SMCs via muscarinic M_3_ receptors.

We chose to focus the current work on *CHRM3* in view of its fundamental biological and medical importance (Wess et al., 2007; Gericke et al., 2011, 2014) but our initial RNA-sequencing analysis suggested upregulation of the muscarinic M_2_ and M_5_ receptors by MYOCD. A common upstream regulator of M_2_ and M_3_ could explain why these receptors are often co-expressed, but we were unable confirm the effect on M_2_ using RT-qPCR. We are uncertain of the explanation for this. One possibility is that the effect of MYOCD on *CHRM2* is smaller than is the effect on *CHRM3*, as suggested by the RNA-sequencing, but technical issues with the human *CHRM2* primer cannot be ruled out. The mouse primer for *Chrm2* appeared to work well in cultured mouse organs and using Srf-deficient tissues, but no reduction was apparent in either case, even if *Chrm3* was reduced. Regulation of M_2_ and M_5_ therefore needs to be investigated further.

To summarize, the current work has identified the muscarinic M_3_ receptor, *CHRM3*, as a target of MRTFs and serum response factor in human SMCs *in vitro* and in the mouse urinary bladder and ileum *in vivo*. Among the MRTFs, MRTF-B appears to be the strongest transactivator of *CHRM3*, consistent with the high expression of MRTF-B in endothelial cells and in keeping with a dominance of endothelium-dependent dilatation over direct SMC-dependent cholinergic vasoconstriction.

## Data Availability Statement

The raw bulk RNA-Seq data is submitted to the Sequence Read Archive with the BioProject PRJNA731342 (https://www.ncbi.nlm.nih.gov/bioproject/PRJNA731342).

## Ethics Statement

The animal study was reviewed and approved by the Malmö – Lunds djurförsöksetiska nämnd.

## Author Contributions

LL, CR, OH, DK, SF, ME, and KS participated in the study design. LL, CR, ME, and KS collected the data. KS generated the funding and wrote the manuscript. All authors were involved in manuscript revisions. All authors read and approved the submitted version.

## Conflict of Interest

The authors declare that the research was conducted in the absence of any commercial or financial relationships that could be construed as a potential conflict of interest.

## Publisher’s Note

All claims expressed in this article are solely those of the authors and do not necessarily represent those of their affiliated organizations, or those of the publisher, the editors and the reviewers. Any product that may be evaluated in this article, or claim that may be made by its manufacturer, is not guaranteed or endorsed by the publisher.
