# Erratum: Regulation of the Muscarinic M_3_ Receptor by Myocardin-Related Transcription Factors

**DOI:** 10.3389/fphys.2021.782588

**Published:** 2021-10-08

**Authors:** Frontiers Production Office

**Affiliations:** Frontiers Media SA, Lausanne, Switzerland

**Keywords:** cholinergic neurotransmission, pharmacology, acetylcholine, signaling, vasodilatation

Due to a production error, Figures 2–7 were erroneously mismatched to their figure legends. The correct figures and their legends appear below.

**Figure 2:**
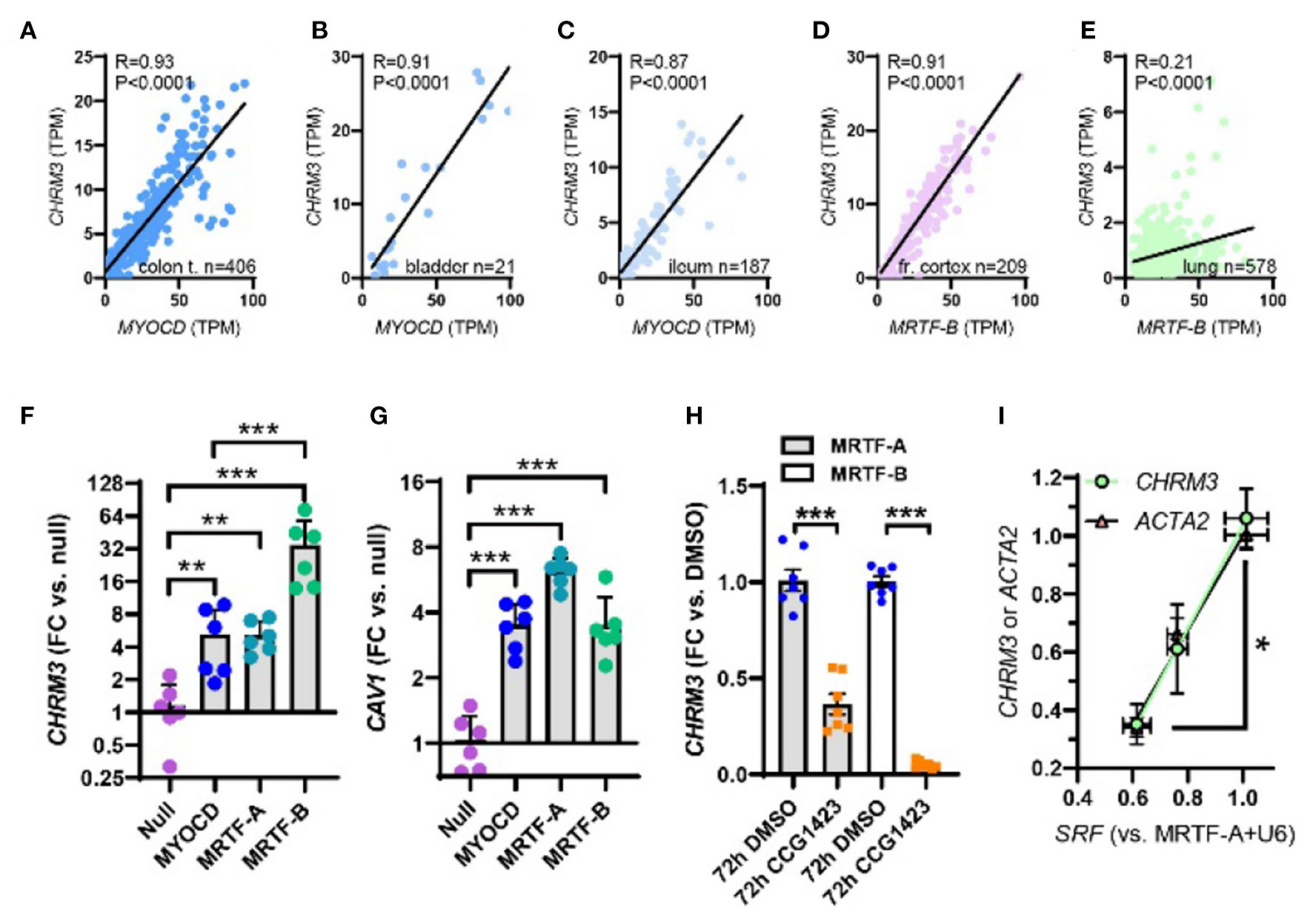
Myocardin (MYOCD) correlates with the M3 muscarinic receptor (*CHRM3*) across human tissues and SRF is critical for *CHRM3* regulation by MRTFs. **(A)** through **(C)** show correlations at the mRNA level of MYOCD vs. M3 (*CHRM3*) in the human gastrointestinal tract and urinary bladder. In brain **(D)** and lung **(E)**, *MRTFB*, rather than *MYOCD*, correlated with M3. This prompted us to examine if all MRTFs (MYOCD, MRTF-A, and MRTF-B) regulate M3 at the mRNA level. Viral overexpression in human coronary artery SMCs showed that MRTF-B was a more effective transactivator of *CHRM3* than MYOCD [**(F)**, *n* = 6], despite having the same effect as MYOCD on another target [*CAV1*, **(G)**]. **(H)** Shows reduction of *CHRM3* after treatment for 72 h with the MRTF-SRF inhibitor CCG-1423 (10 μM, *n* = 6). Cells were transduced with either MRTF-A (gray bars) or MRTF-B (white bars). **(I)** Shows that knockdown of serum response factor (SRF, 0, 30, and 100 MOI of Ad-shSRF) reduces *CHRM3* (green/black circles) in parallel with *ACTA2* (pink/black triangles, *n* = 4, per condition). MRTF-A was overexpressed throughout in **(I)**. ***p < 0.001, **p < 0.01, and *p < 0.05.

**Figure 3:**
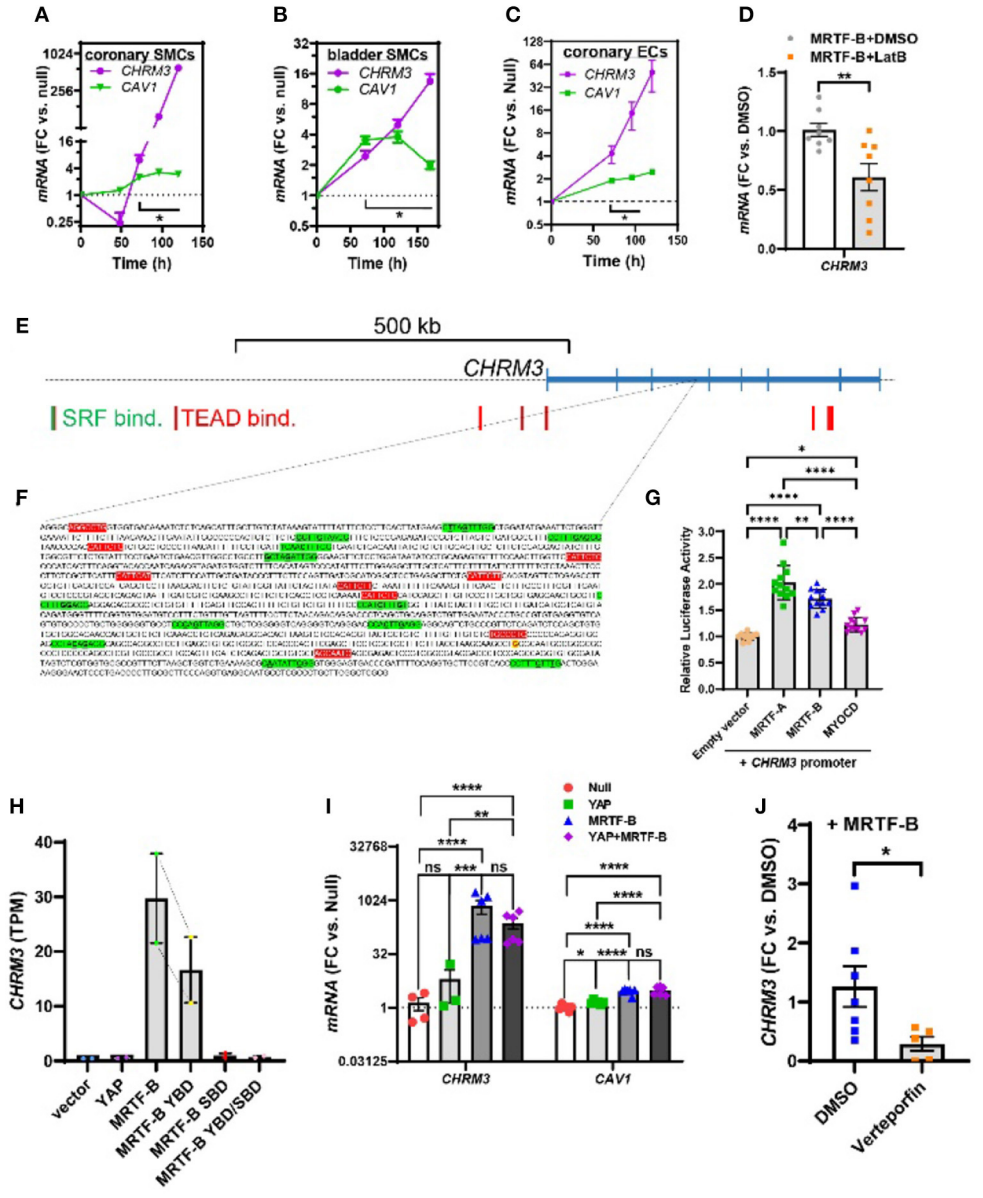
Time-course data, promoter reporter assays, and MRTF-B-YAP cooperation. MRTF-B was overexpressed in different cell types (200 MOI), and cells were harvested at different times. RNA was subsequently isolated, and *CHRM3* was measured by RT-qPCR. **(A)** Shows time-dependent upregulation of *CHRM3* in human coronary artery SMCs. Significant increases were seen at times exceeding 48 h, and a 597-fold increase was seen at 120 h (*p* = 0.0004, *n* = 3). Because there was no indication that the increase of *CHRM3* reached a plateau at longer transduction times, we designed an experiment using even longer incubations in human bladder SMCs **(B)**. Again, there was no tendency of a plateau. Moreover, the maximal increase was somewhat smaller than in coronary artery SMCs. Similar results were obtained in human coronary artery endothelial cells [**(C)**, 200 MOI]. **(D)** Shows that *CHRM3* was reduced by Latrunculin B (100 nM, gray bar) in MRTF-B-transduced ECs. Inspection of the *CHRM3* gene locus on chromosome 1 **(E)** revealed SRF binding (green vertical bars) and TEAD binding (red vertical bars) to many 5' sequences of, and over, the longest transcript (blue). Direct examination of a commercial promoter reporter sequence (NM_000740, transcript variant 2, hg38; chr1+: 239,627,686–239,629,364; TSS = 239,629,073) did not reveal any true CArGs, but 11 motifs with 2 deviations from the classical CArG sequence [CC(A/T)6GG, green highlights, deviations underlined, **(F)**] were present, along with 9 TEAD motifs [red highlights, **(F)**]. The transcription start site for the promoter is highlighted in yellow with red lettering. This “CArG-deficient” promoter responded to MRTFs in a luciferase reporter assay **(G)** run using HEK 293 cells. **(H)** Shows *CHRM3* mRNA expression in MCF10 cells transfected with YAP, MRTF-B, and two MRTF-B mutants; the YBD mutant does not bind YAP, and the SBD mutant does not bind SRF. **(I)** Shows the effects of YAP and MRTF-B transduction, alone and in combination, on *CHRM3* in human coronary artery SMCs. Ct values for *CHRM3* were sometimes too high for reliable detection (null and YAP). This is the reason why the sample size is less than *n* = 6 for *CHRM3* in the null and YAP groups, even if six experiments were run for the panel. **(J)** Shows the effect of the YAP-TEAD inhibitor verteporfin in MRTF-B-transduced coronary artery SMCs. Two samples were lost in the verteporfin group again due to lack of amplification. ****p < 0.0001, ***p < 0.001, **p < 0.01, and *p < 0.05.

**Figure 4:**
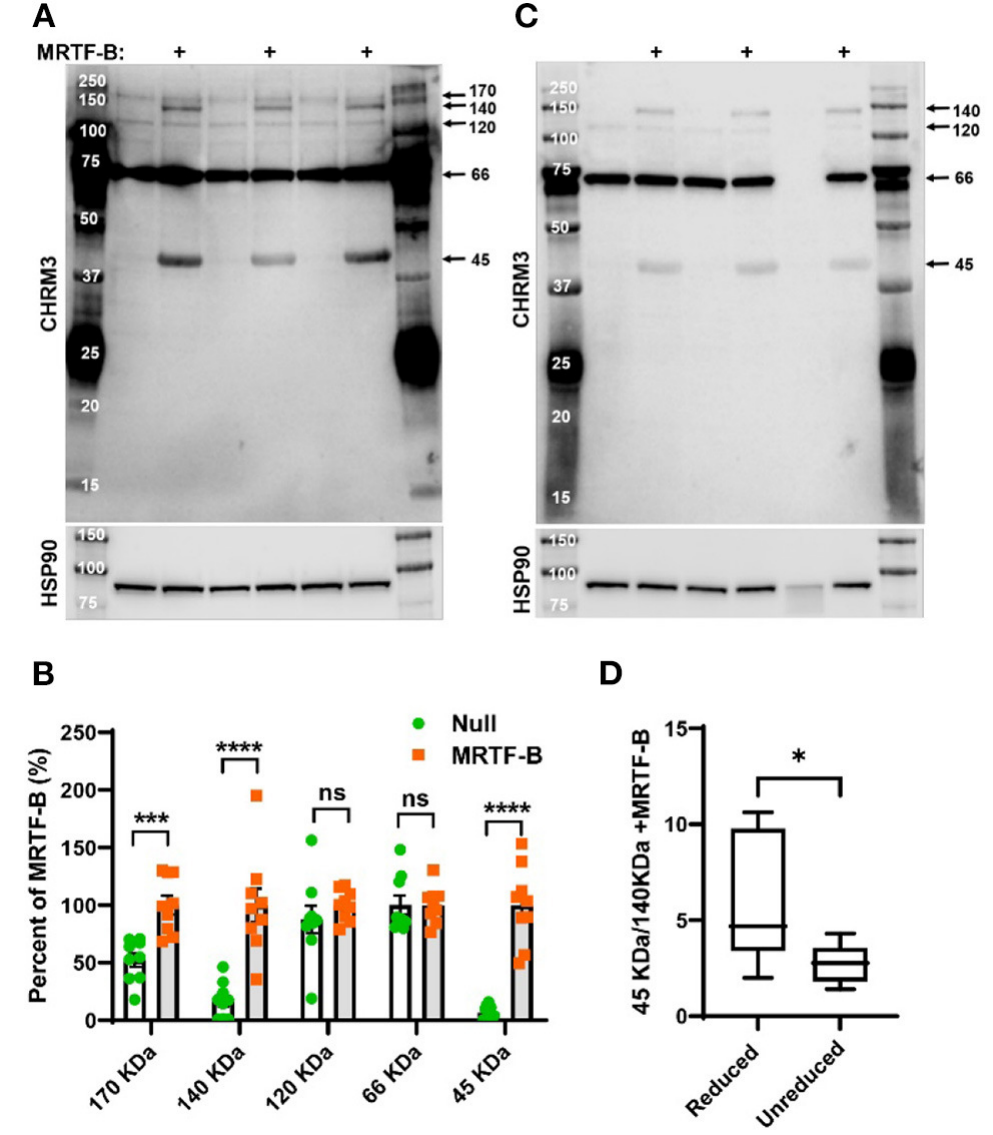
MRTF-B increases CHRM3 immunoreactive bands in human coronary artery smooth muscle cells. Protein lysates from human coronary artery SMCs treated with null virus and with MRTF-B virus for 120 h, respectively, were used for Western blotting using an antibody raised against a human CHRM3 in **(A)**. After developing the blot, it was stripped and incubated with HSP90 antibody (shown below) to assess equal protein loading. At least five bands were detected with the CHRM3 antibody, and three bands at ≈170, ≈140, and ≈45 kDa changed significantly with MRTF-B as shown in the compiled analysis in **(B)**. Quantification in **(B)** was done using the blots in **(A)**. Proteins from the same original lysates were also prepared in non-reducing conditions, to examine if the relationship between bands changed **(C)**. The volume was insufficient for one of the null samples in this experiment (lane 6). Non-reducing conditions favored the 140 kDa band at the expense of the 45 kDa band **(D)**, suggesting multimerization. ^****^p < 0.0001, ^***^p < 0.001, and ^*^p < 0.05.

**Figure 5:**
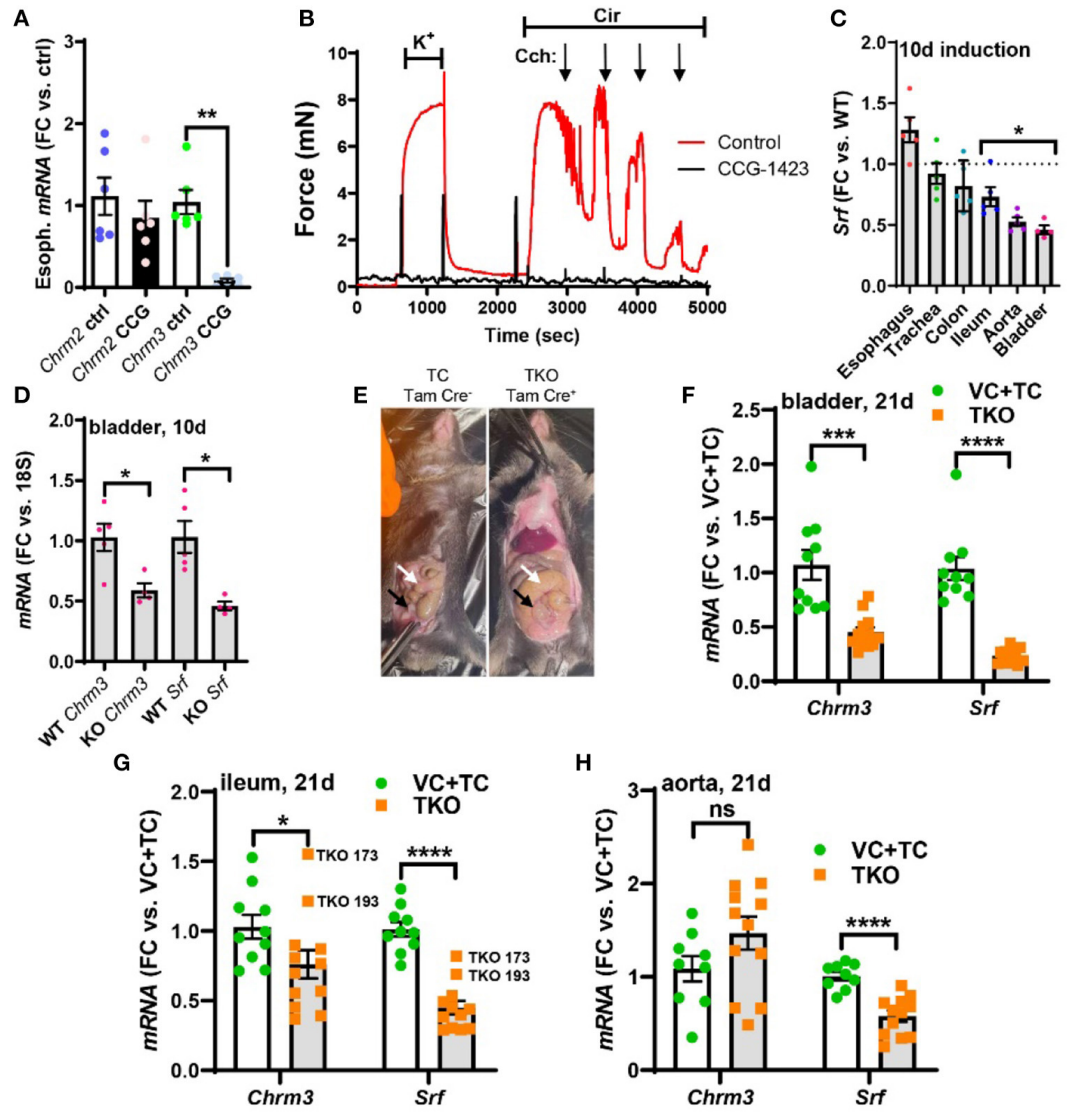
Pharmacological inhibition of MRTF-SRF signaling in organ culture, and knockout of Srf *in vivo*, reduces M3 receptor expression. To examine if MRTF-SRF signaling regulates muscarinic M3 receptor expression *in situ*, we first isolated organs from wild-type C57Bl/6 mice. Organs were split in half and maintained for 96 h in organ culture with vehicle (DMSO) or CCG-1423 (10 μM). A clear reduction of *Chrm3* relative to the house-keeping gene *18 s* was seen in the esophagus **(A)**, but in the remainder of the organs, the house-keeping genes examined declined (not shown). We also found that organ culture of the mouse caudal artery in the presence of CCG-1423 eliminated force development on stimulation with the α1-adrenergic agonist cirazoline **(B)**, suggesting that this experimental paradigm is unsuitable for studying effects on endothelium-dependent dilatation. Mice with SMC-specific knockout of Srf were next obtained by injecting Srffl/fl mice harboring the Myh11-Cre/ERT2 transgene with tamoxifen for 5 consecutive days (knockout: KO). Cre-negative Srffl/fl mice injected with tamoxifen were used as controls (wild-type: WT). Organs were harvested and frozen 10 days after the first injection and transcript levels were determined by RT-qPCR. At this time, body weights were unchanged, but Srf depletion was seen in some organs **(C)**. **(D)** Shows that *Chrm3* was reduced in parallel with *Srf* in the bladder, but this was not seen elsewhere (not shown). We therefore next used mice at 21 days post tamoxifen. Two control groups were included in this second experiment along with the tamoxifen-treated knockouts (TKO): vehicle-treated Cre-positive mice (VC) and tamoxifen-treated Cre-negative mice (TC). At 21 days, mobility on provocation was reduced, the intestines had started to swell [**(E)**, white arrows], and the urinary bladders were often enlarged [**(E)**, black arrows]. Both *Chrm3* and *Srf* were reduced in the bladder **(F)** and ileum **(G)**. For the ileum, the two knockouts with the most modest *Srf* depletion (TKO 173 and TKO 193) are highlighted. No change of *Chrm3* was seen in the aorta **(H)**, despite significant *Srf* depletion. These findings show that MRTF-SRF signaling is critical for *Chrm3* expression in gastrointestinal and urogenital organs *in vivo*. ***p < 0.001, **p < 0.01, and *p < 0.05.

**Figure 6:**
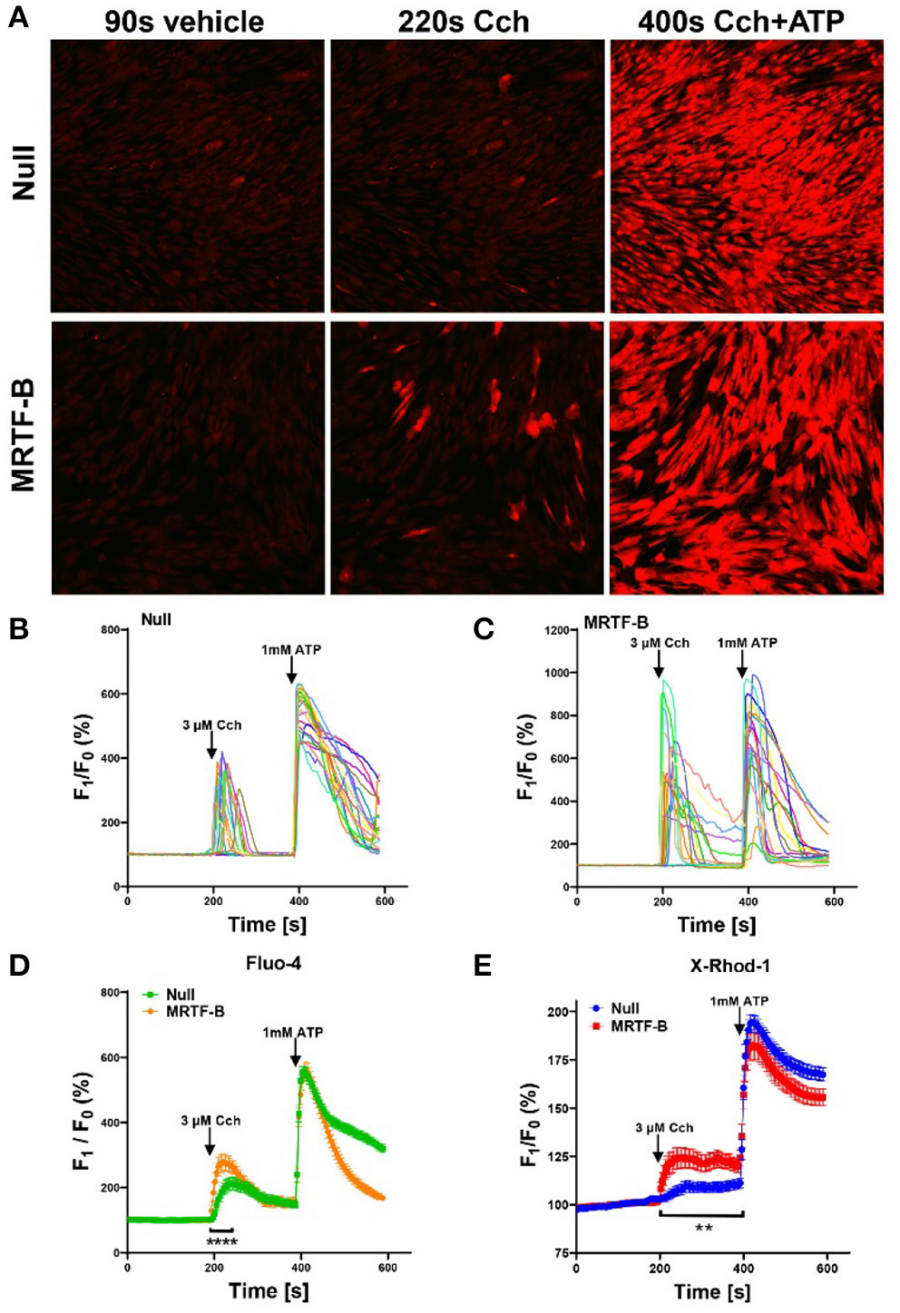
Ca2+ imaging of MRTF-B-transduced human coronary artery SMCs. To examine if MRTF-B transduction increases responsiveness to the muscarinic agonist carbachol, cells were treated as indicated for 5 days in culture. They were subsequently washed and loaded with Fluo-4 and imaged using confocal microscopy. Only a fraction of the cells responded to carbachol [**(A)**, middle], but the responses were larger in MRTF-B-transduced cells. **(B**, **C)** Show the 20 cells responding best to carbachol in the experiment in **(A)**. **(D)** Shows compiled data from three independent experiments with Fluo-4. The Ca2+ signal between 200 and 250 s was significantly increased by prior MRTF-B transduction. **(E)** Shows intracellular Ca2+ in human coronary artery SMCs after transduction of MRTF-B or null virus, respectively (*N* = 6), but measured Ca2+ using X-Rhod-1. Error bars in **(D,E)** represent 95% confidence intervals. ****p < 0.0001 and **p < 0.01.

**Figure 7:**
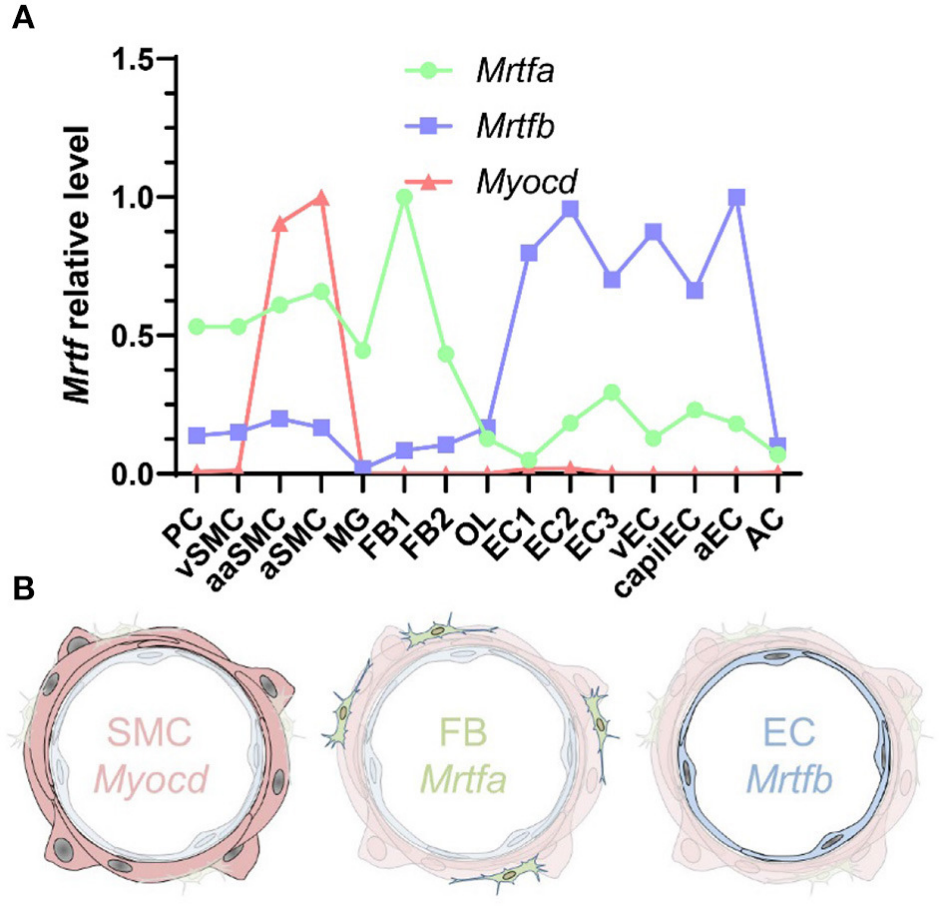
MRTF distribution across the vascular wall. **(A)** Shows cell-averaged mRNA expression data for the indicated transcripts in different cerebrovascular cell types. PC, pericytes; SMC, smooth muscle cells; MG, microglia; OL, oligodendrocytes; FB, fibroblasts; EC, endothelial cells; AC, astrocytes; v, venous; cap, capillary; a, arterial; aa, arteriolar; and 1, 2, and 3, subtype. **(B)** Shows a schematic representation of Myocd in SMCs, Mrtfa in FBs, and Mrtfb in ECs.

The publisher apologizes for this mistake. The original article has been updated.

